# Neuromorphic Floating-Gate Memory Based on 2D Materials

**DOI:** 10.34133/cbsystems.0256

**Published:** 2025-04-22

**Authors:** Chao Hu, Lijuan Liang, Jinran Yu, Liuqi Cheng, Nianjie Zhang, Yifei Wang, Yichen Wei, Yixuan Fu, Zhong Lin Wang, Qijun Sun

**Affiliations:** ^1^ School of Printing and Packaging Engineering, Beijing Institute of Graphic Communication, Beijing 102627, P. R. China.; ^2^ Beijing Institute of Nanoenergy and Nanosystems, Chinese Academy of Sciences, Beijing 101400, P. R. China.

## Abstract

In recent years, the rapid progression of artificial intelligence and the Internet of Things has led to a significant increase in the demand for advanced computing capabilities and more robust data storage solutions. In light of these challenges, neuromorphic computing, inspired by human brain’s architecture and operation principle, has surfaced as a promising answer to the growing technological demands. This novel methodology emulates the biological synaptic mechanisms for information processing, enabling efficient data transmission and computation at the identical position. Two-dimensional (2D) materials, distinguished by their atomic thickness and tunable physical properties, exhibit substantial potential in emulating synaptic plasticity and find broad applications in neuromorphic computing. With respect to device architecture, memory devices based on floating-gate (FG) structures demonstrate robust data retention capabilities and have been widely used in the realm of flash memory. This review begins with a succinct introduction to 2D materials and FG transistors, followed by an in-depth discussion on remarkable research progress in the integration of 2D materials with FG transistors for applications in neuromorphic computing and memory. This paper offers a thorough review of the existing research landscape, encapsulating the notable progress in swiftly expanding field. In conclusion, it addresses the constraints encountered by FG transistors using 2D materials and delineates potential future trajectories for investigation and innovation within this area.

## Introduction

**Fig. 1.**
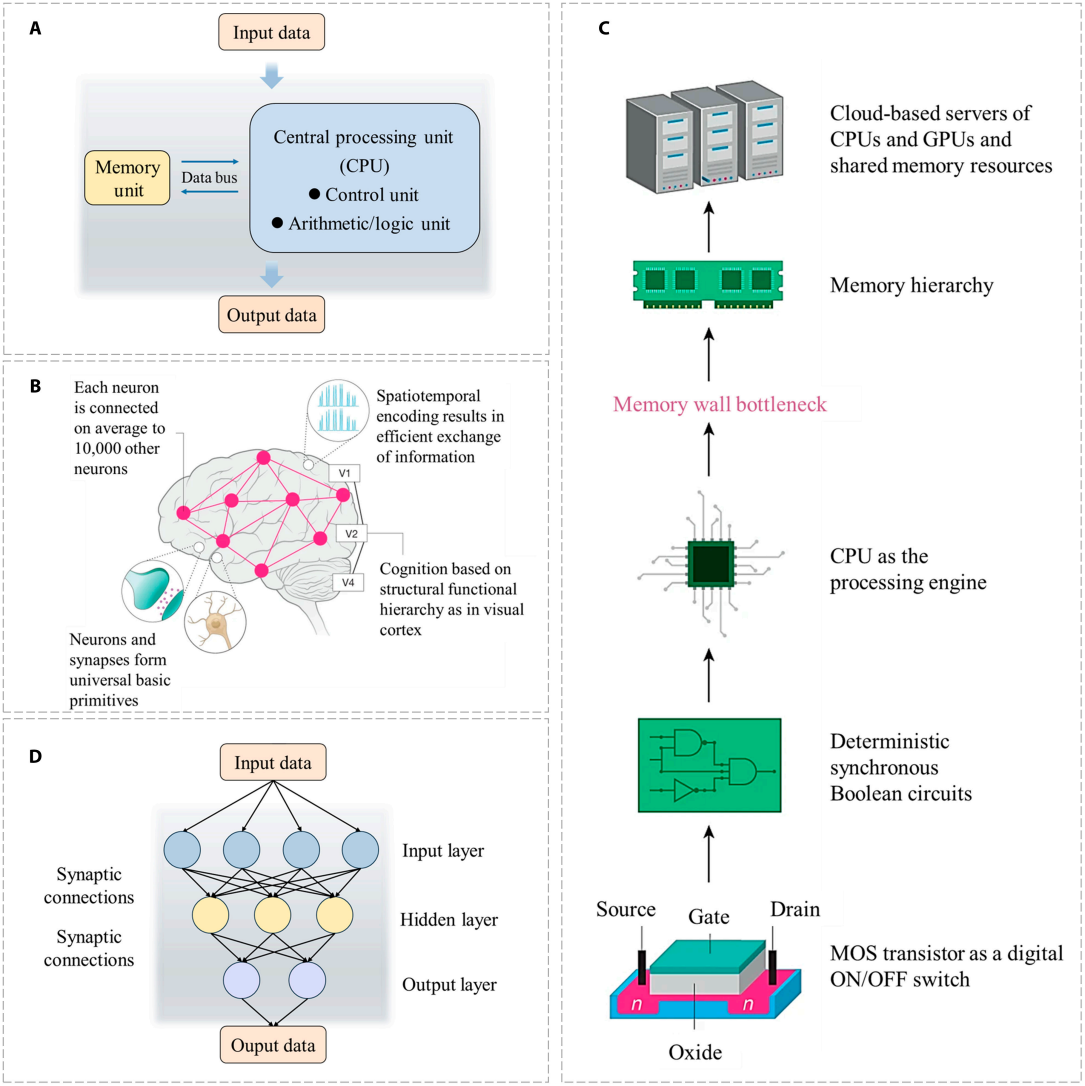
Contrast between the von Neumann architecture and neuromorphic computing. (A) von Neumann architecture. (B) A diagram illustrating the fundamental organizational principles of the brain [8]. (C) A state-of-the-art Si computing system [8]. (D) Neuromorphic computing.

Utilizing metal–oxide–semiconductor field-effect transistors (MOSFETs) as a critical component within the framework of integrated circuits, computer technology has firmly established a foundation for the contemporary-information-driven society. This has led to groundbreaking advancements across a multitude of technological domains. Nevertheless, the distinct separation of the memory’s physical structure and the central processing unit (CPU) necessitates that computers execute tasks in a linear sequence. When handling extensive amounts of data, the information transfer between the processor and the memory results in substantial power consumption and decreased computational speed, which substantially impedes the computational capability (i.e., the von Neumann bottleneck) (Fig. 1A) [1–3]. To augment the performance of traditional computers, it is imperative to integrate a greater number of processors and memories into a single chip within a specified area or volume. However, the scaling challenges associated with processors and memories made from silicon transistors are becoming increasingly pronounced because of the constraints imposed by Moore’s law. To realize reduced power consumption and enhanced data processing efficiency, it is imperative to design novel computing architectures leveraging innovative materials and fundamental principles.

Neuromorphic computing, inspired by the human brain’s capacity for parallel computation and adaptive learning, has been raised as a solution to the inherent von Neumann bottleneck in traditional computing frameworks, leading to a series of technology breakthroughs [4]. The human brain’s nervous system, composed of approximately 10^10^ to 10^12^ neurons, is intricately linked through synapses with each connected to hundreds of others, thereby enabling the transmission and reception of signals. The brain, with a power consumption of only ~20 W, is responsible for categorizing stimuli, predicting outcomes, generating thoughts, and ensuring vital life functions [5]. Conversely, although contemporary digital computers are proficient at executing high-precision calculations, they necessitate substantial energy consumption when performing cognitive tasks, which are the domain of the brain’s expertise. For instance, the energy required to train the most advanced natural language processing models on contemporary supercomputers amounts to 1,000 kWh of energy [6], equivalent to the energy that the human brain would expend over 6 years performing all tasks. While our understanding of the brain remains incomplete, its remarkable capabilities can be ascribed to 3 key aspects explored in neuroscience: the vast connectivity, the organized functional hierarchy, and the temporally dependent functions of neurons and synapses (Fig. 1B) [7]. Contemporary deep learning networks are fundamentally inspired by the brain’s hierarchical structure and synaptic framework, comprising multiple layers or transformations, each representing distinct latent features within the input. The operation of these neural networks is facilitated by hardware computing systems, fundamentally reliant on silicon-based FETs. The digital logic used for large-scale computing is composed of over billions of transistors, all integrated onto a single silicon chip. Various stages of silicon-based transistor computation are strategically layered to optimize data transfer patterns (Fig. 1C) [8]. Although this computing model bears a superficial resemblance to neuromorphic computing, it fundamentally operates on the conventional von Neumann architecture. The physical separation between processing units and the memory devices intensifies the “memory wall bottleneck” [9], leading to inefficiencies and increased power consumption due to the CPU’s idle waiting for data from slower memory components.

Traditional software or hardware strategies to realizing neuromorphic computing have challenges of high power consumption, low integration density, and insufficient reliability, which urgently necessitates to further advance neuromorphic/brain-like computing with new materials or architectures. In 1990, Mead [10] first used the term “neuromorphic” to characterize the transmission and processing of neural information in high efficiency, which differs from traditional digital computing system. Neuromorphic computing simulates the information processing behaviors in biological synapses (core physical mechanism: ion migration through synapses), enabling efficient information transmission and processing at the same position. As shown in Fig. 1D, neuromorphic computing enables dual functions of information storage and computation at the same position, eliminating the need for additional data transmission. Its advantages include lower energy dissipation (in nanojoules) and faster processing speed (in nanoseconds) [11]. Moreover, neuromorphic computing features distributed memory and computational components [12], which can facilitate efficient, large-scale parallel computing to overcome the von Neumann bottleneck.

To propel brain-like neuromorphic computing through material innovation, researchers have identified 2-dimensional (2D) materials as a pivotal candidate. These materials exhibit exceptional potential due to their atomic thickness, clean surfaces devoid of dangling bonds, unique physical properties, electrical tunability [13–15], and high integrability. Such intrinsic advantages position 2D materials as key feasible solutions for neuromorphic computing. In addition, the inherent high surface-area-to-volume ratio, along with sensitivity to charge transfer and electrostatic modulation at interfaces, renders 2D materials an optimal platform for fabricating synaptic devices with reliable operational principles [16,17].

In terms of device architecture, floating-gate (FG) transistors, commonly used as the basic structure in flash memory devices such as NOR and NAND flash [18,19], have attracted attention due to their advantages in miniaturization, low energy consumption, and high-efficiency data storage, which effectively address the challenges related to high-density integration and large-capacity data processing. Meanwhile, FG memory devices based on the principle of charge storage have the potential to emulate synaptic plasticity, potentially breaking through the von Neumann computing architecture and serving as artificial synaptic units inspired by the human brain [20–29]. Traditional FG memory is based on the MOSFET structure, where the storage function is facilitated by incorporating an FG layer to capture and release carriers [30–33]. When a proper voltage is applied to the gate of the FG transistor, charge injection/removal occurs in the FG layer, known as programming/erasing operations. On the basis of the charge capture/release process in the FG layer, nonvolatile memory behavior can be achieved even in the absence of applied electric field [34]. However, with increased process nodes in computer and decreased transistor size, traditional FG memory devices face issues such as increased leakage current, serious charge leakage, and reduced reliability [35]. For instance, when the thickness of the tunnel oxide layer in FG devices decreases, the stored charges in the FG layer are more prone to leakage, resulting in data instability and reduced memory reliability [36]. Given the atomic-level thickness, high electron mobility, and tunability of 2D materials [37–44], FG transistors incorporating 2D materials exhibit significance potential for neuromorphic computing and advanced memory applications [45–51].

**Fig. 2.**
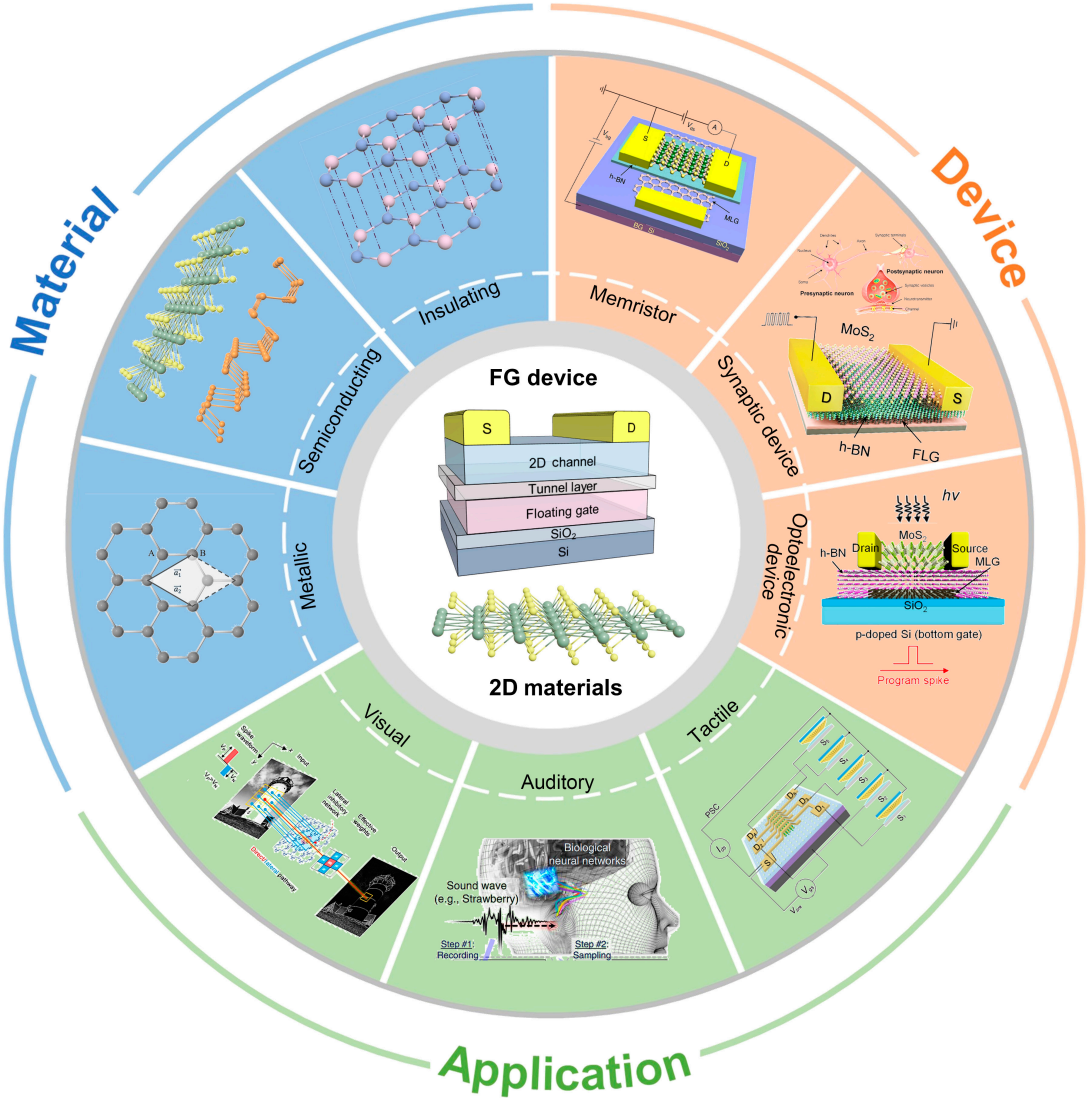
An overview of FG devices integrating 2D materials and their neuromorphic computing applications. MLG, multilayer graphene; PSC, postsynaptic current; BG, bottom gate; FLG, few-layer graphene.

Here, we present a comprehensive overview on the integration of 2D materials with FG transistors for neuromorphic computing and memory applications. To facilitate a deeper understanding of their architectures, we first briefly introduce commonly used 2D materials, including graphene, transition metal dichalcogenides (TMDCs), black phosphorus (BP), and hexagonal boron nitride (h-BN), and analyze their structural differences and unique physicochemical properties such as electron mobility, bandgap, flexibility, and mechanical characteristics. The potential application of these materials in FG transistors is also explored. Then, we introduce the charge tunneling mechanism of FG transistors and summarize the development of FG transistors classified by 5 common structures. In addition, we systematically review various 2D FG devices and their applications in memory devices and neuromorphic computation in terms of materials, devices, and applications (Fig. 2). Finally, we raise the issues of 2D FG transistors related to material preparation, device structure design, device stability control, and integration and outline the potential development trends for exploration and innovation within this domain.

## Fundamentals of 2D Materials and FG Transistors

### Fundamentals of 2D materials

Since the exfoliation of graphene in 2004 [52], the diversity of 2D materials has been continuously expanding, opening up a vast research field [53–57]. 2D materials, i.e., van der Waals (vdW) materials, are featured by strong covalent bonds within individual layer and weak vdW forces across layers. When bulk crystals are exfoliated layer by layer, the sheets can maintain their structural integrity down to a single layer and can effectively suppress short-channel effects. The multifunctionality of 2D materials is exciting, including conductors, semimetals, semiconductors with adjustable bandgaps, and insulators [58–64]. Among the reported 2D materials, graphene, BP, h-BN, and TMDCs stand out because of their stable chemical properties, good thermal performance, and complementary features. These materials play a crucial role in future developments in integrated circuits [65], neuromorphic computing [66–73], and other fields.

#### Graphene

**Fig. 3.**
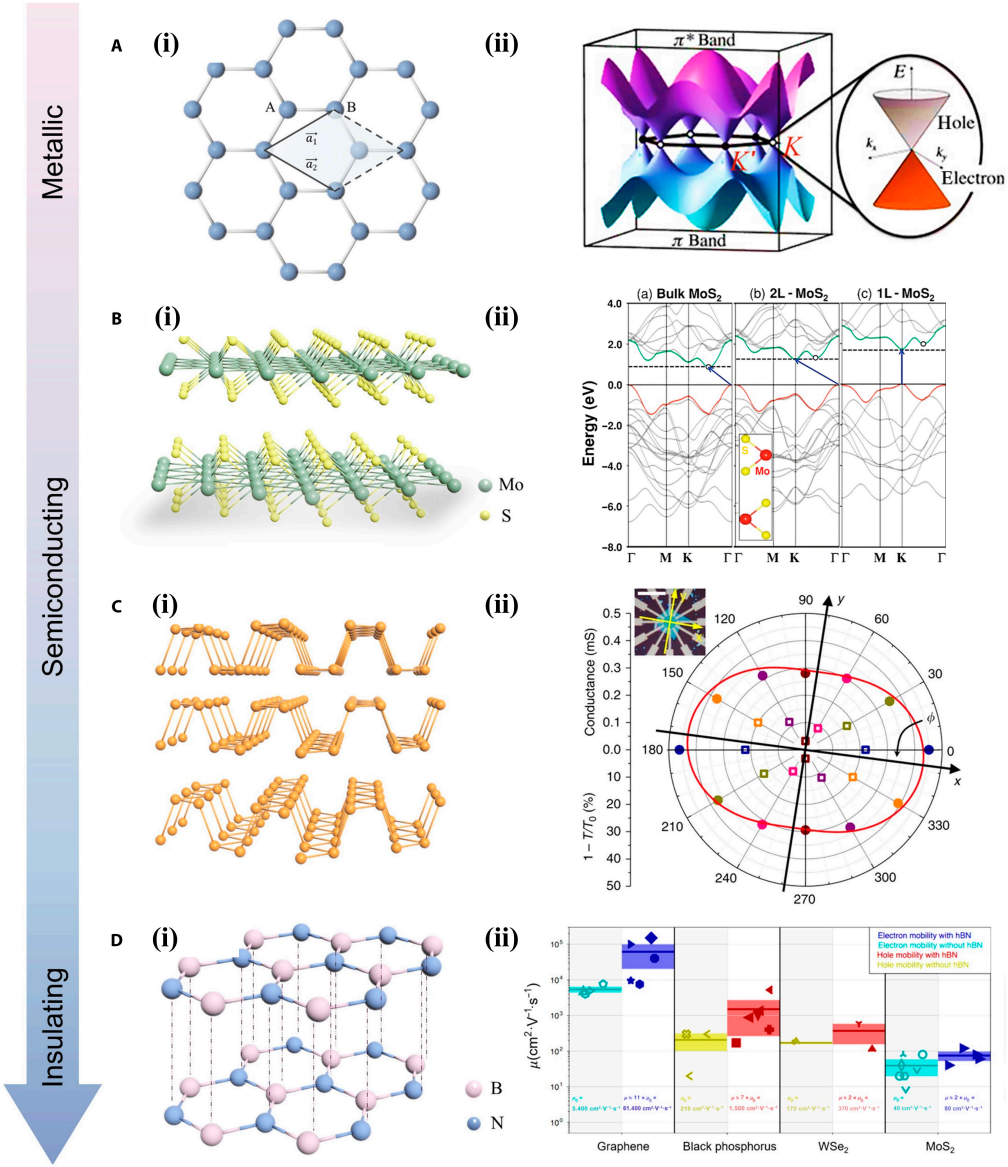
Distinct characteristics of common 2D materials. (A) (i) A diagram of graphene lattice structure, where A and B represent the 2 distinct carbon atoms. The unit cell is highlighted in the shaded area, and $\vec{a_{1}}$, $\vec{a_{2}}$ are the basis vectors in real space [74]. (ii) The electronic band structure of Gr showing a zero bandgap [80]. (B) (i) Schematic crystal lattice structure of MoS_2_. (ii) The dependence of band structures on thickness for bulk, bilayer, and monolayer MoS_2_ [98]. (C) (i) Schematic crystal lattice structure of BP. (ii) The dc conductivity and infrared relative extinction measured along the same 6 directions [106]. (D) (i) Schematic crystal lattice structure of h-BN. (ii) Mobility in h-BN heterostructures [113].

Graphene consists of a single atomic layer of carbon atoms organized in a hexagonal honeycomb structure, with each unit cell containing 2 inequivalent atoms, labeled as A and B, defined by the basis vectors ($\vec{a_{1}}$, $\vec{a_{2}}$). As shown in Fig. 3A-i [74], 3 of the carbon electrons form strong in-plane bonds through sp^2^ hybridization, while the remaining *P*_z_ orbital electrons endow graphene with excellent electronic and optical properties. Graphene’s structure is highly stable, with carbon–carbon bonds measuring only 1.42 Å in length [75]. The bond between carbon atoms within graphene is flexible; when subjected to external forces, the carbon plane deforms and bends, enabling carbon atoms to adjust without rearrangement, thus maintaining structural stability. In addition, as electrons traverse the orbitals in graphene, they do not scatter caused by lattice imperfections or the presence of foreign atoms [76–79]. Owing to the strong atomic interactions, even when neighboring carbon atoms collide at room temperature, the disturbance to electrons within graphene remains minimal. Monolayer graphene exhibits a zero-bandgap structure (Fig. 3A-ii), indicating that it is conductive regardless of where the Fermi level is situated [80], leading to a very high electron mobility and making it a perfect material for applications in high-frequency devices [81]. The limited density of states and unique band structure of graphene allow for easy modulation of the Fermi level with an external electric field, thus enabling graphene to exhibit ambipolar field-effect transport properties [82,83]. In the research of 2D FG devices, graphene is widely used as the FG layer due to its excellent electron mobility, large surface area, outstanding electrical conductivity, superior thermal conductivity, and high mechanical strength [20,24,25,29,84–95].

#### Transition metal dichalcogenides

The TMDC family is extensive and expanding, with a common chemical formula MX_2_, where M represents a transition metal and X denotes a chalcogenide element. For instance, Fig. 3B-i illustrates the crystal structure of a representative TMDC material, MoS_2_. Similar to graphene, monolayer MoS_2_ can be fabricated through mechanical exfoliation, facilitated by the weak interlayer vdW bonds [96]. TMDCs commonly exhibit different structural phases, primarily the trigonal prismatic phase (2H) and the octahedral phase (1T). The various structural forms of single-layer TMDCs essentially result from distinct stacking configurations of the 3 atomic layers (chalcogen–metal–chalcogen), where the 2H phase is of ABA-type stacking with chalcogen layers aligned vertically and the 1T phase is of ABC-type stacking. For compounds composed of different transition metal groups and chalcogen elements, either the 2H or 1T phase is the thermodynamically stable phase. In general, when the 2H phase is stable, the 1T phase is metastable, and vice versa. WTe_2_ is an exception, as its stable phase is the orthorhombic 1T phase at room temperature. For multilayer or bulk TMDCs, their structural description is usually based on the stacking method of single-layer TMDCs, although it might be affected by distortions. Strong distortions can result in the formation of metal–metal bonds, causing the transition from the 1T phase to the 1T′ phase in group VI TMDCs; lighter distortions may result in the emergence of charge density wave phases [54]. TMDCs showcase a wide range of bandgap variations, including materials with no bandgap (such as WTe_2_ and TiS_2_) [97], making them range from insulators (such as Mo and W dichalcogenides) to metallic or semimetallic materials (such as V and Nb dichalcogenides). The layer number has a considerable impact on the bandgap of TMDCs. As shown in Fig. 3B-ii, MoS_2_ exhibits a transition from the indirect bandgap in its multilayer form (1.29 eV) to the direct bandgap in its monolayer form (1.80 eV) [98]. The bandgaps corresponding to the visible and near-infrared spectrum [99,100] make TMDCs highly sought-after materials for optical and optoelectronic devices operating within the visible and near-infrared bands [59,82,101].

#### Black phosphorus

BP, another typical layered 2D semiconductor, has garnered extensive research interest. Fig. 3C-i displays the crystal structure of BP, which differs from graphene and TMDCs in that its direct bandgap ranges from 0.35 eV in bulk material to 1.7 eV in a single layer [102,103]. The narrow direct bandgap of BP is located between that of graphene (zero bandgap) and TMDCs (>1-eV bandgap), with their spectral wavelength covering visible light to mid-infrared. This property is crucial for developing various optoelectronic technologies, e.g., remote communication, sensors, and solar cells [104]. Moreover, this energy range aligns with the bandgaps of current semiconductor technologies, such as silicon and III–V semiconductors, offering considerable research and application potentials for materials with this bandgap [105]. Because of its ability to detect infrared light, BP is regarded as ideal for polarized light detection. Furthermore, the low crystal symmetry of BP results in notable in-plane carrier transport anisotropy properties (Fig. 3C-ii), making it a promising material for multifunctional optoelectronic devices [106].

#### Hexagonal boron nitride

As the most commonly encountered 2D insulating material, h-BN is widely regarded as excellent gate dielectrics for 2D FETs. As shown in Fig. 3D-i, h-BN features a covalently bonded hexagonal structure composed of alternating nitrogen and boron atoms. Each boron atom forms covalent bonds with 3 nitrogen atoms, and each nitrogen atom is similarly bonded to 3 boron atoms, resulting in a honeycomb lattice structure [107]. However, unlike the carbon atoms in graphene, h-BN is composed of 2 different elements, resulting in different electronic properties and potential applications. The lattice constant of h-BN is slightly higher than graphene, allowing the 2 materials to be stacked together to form vdW heterostructures [108]. The bond energy between B and N is very high, reaching about 4 eV, which is higher than the 3.7 eV of the C–C bonds in graphene. The high bond energy endows h-BN with exceptional chemical and thermal stability [109]. In addition, h-BN possesses an extremely high intrinsic tensile strength and elastic modulus, making it outstanding in terms of mechanical performance [110]. h-BN with high dielectric constant used for gate insulator is typically due to its wide bandgap of approximately 5 eV, which renders it nonconductive [111]. Its low thermal expansion coefficient and high thermal conductivity are similar to stainless steel and second only to quartz glass, which is highly suitable as an insulating and supporting material in high-temperature electronic devices [112]. In addition, a major advantage of h-BN as the gate insulator is its formation of clean vdW interfaces with 2D semiconductors [113]. Commonly, the electron transport in ultrathin 2D semiconductors is highly sensitive to the interface effects and surrounding environment. The roughness of widely used SiO_2_ substrate not only influences the morphology of 2D semiconductors but also markedly changes their charge density and bandgap due to the probably trapped charges in SiO_2_[114]. If h-BN is used instead of SiO_2_, the atomically flat vdW interface can efficiently reduce charge disorder within the system. This configuration minimizes external factors that contribute to charge carrier scattering, such as surface roughness, as well as scattering from charged impurities and remote phonons [113]. Thus, h-BN is highly suitable to be used as gate dielectrics to boost increased carrier mobility in h-BN heterostructures [113]. Beyond its role as a substrate, h-BN has diverse applications in various fields; multilayer h-BN-based capacitors exhibit clear resistive switching behavior, making them suitable for use in resistive random-access memory or use as solid-state synapses within neuromorphic systems [115]. Its high mechanical strength and stretchability also hold promise for flexible gate dielectrics [116]. When the h-BN is in an amorphous form, its dielectric constant could be further decreased, enhancing its suitability as an ideal material for interconnect isolation [117].

### Fundamentals of FG transistor

#### Principles and history of FG transistor

**Fig. 4.**
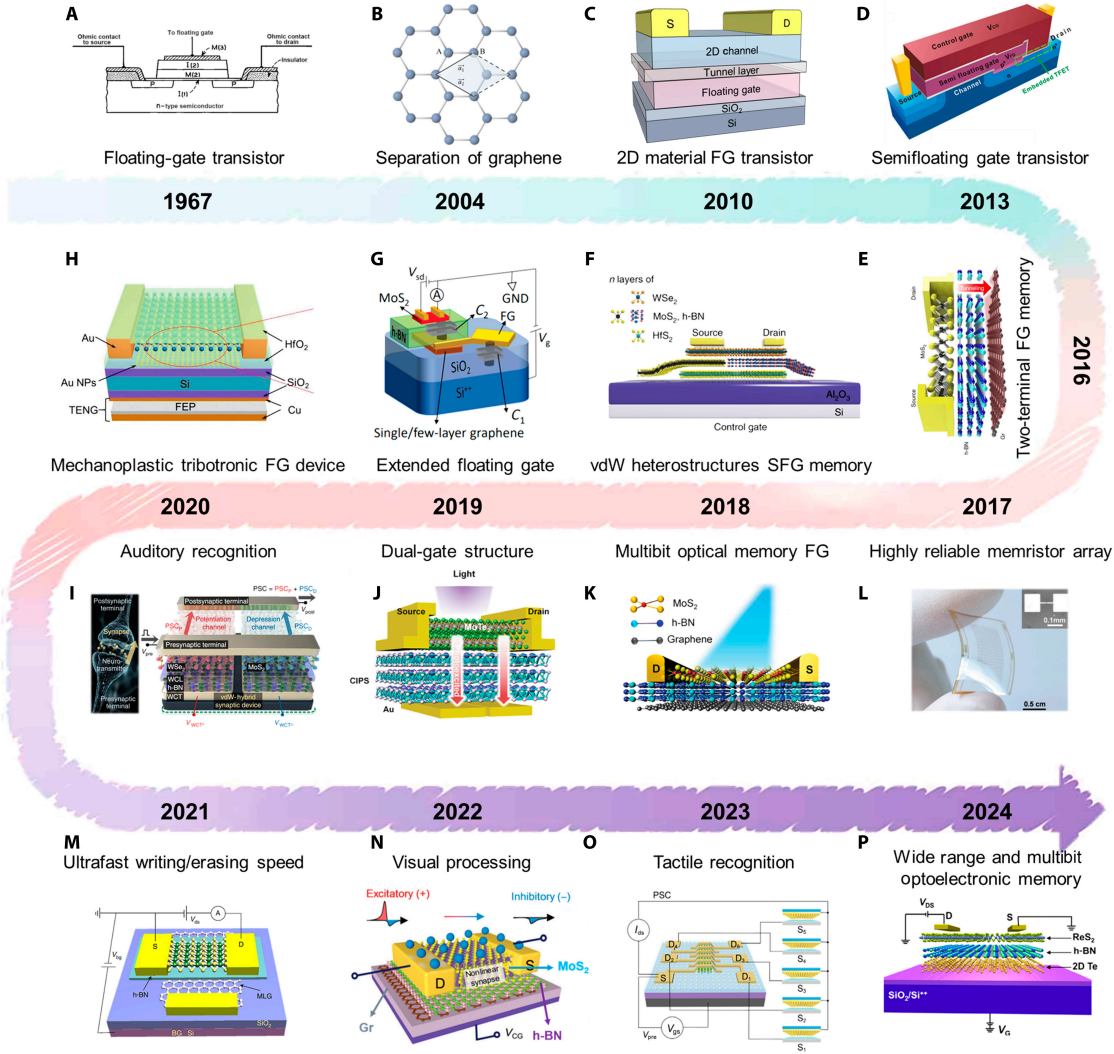
History of the development of FG devices. (A) Floating gate transistor [118]. (B) Separation of graphene [52]. (C) 2D materials used in FG transistors. (D) SFG transistor [141]. TFET, tunneling field effect transistor. (E) Two-terminal FG device [84]. (F) vdW heterostructures SFG device [142]. (G) Extended FG device [208]. GND, ground. (H) Mechanoplastic tribotronic FG device [27]. NPs, nanoparticles. (I) FG device used for auditory recognition [26]. (J) Dual-gate structure FG device [146]. CIPS, CuInP_2_S_6_. (K) Multibit optical memory FG device [88]. (L) Highly reliable memristor array [233]. (M) Ultrafast writing/erasing speed FG device [91]. (N) FG device used for visual processing [29]. (O) FG device used for tactile recognition [23]. (P) Wide range and multibit optoelectronic memory FG device [143].

**Fig. 5.**
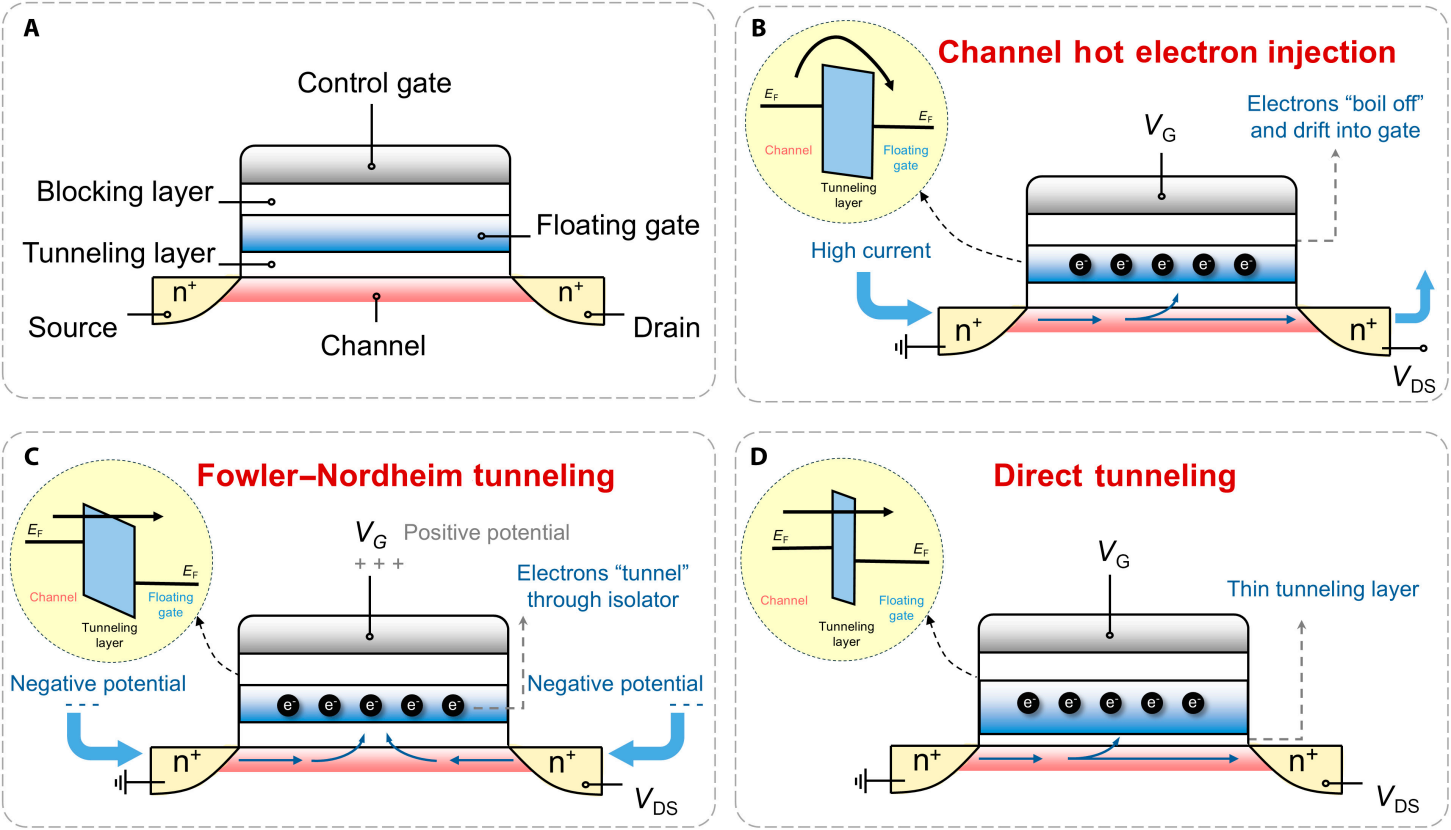
Carrier capture and release mechanism in FG device. (A) Components of an FG device. (B) The principle of channel hot electron injection. (C) The principle of Fowler–Nordheim tunneling. (D) The principle of direct tunneling.

In 1967, Kahng and Sze [118] first utilized metal FG in MOSFETs at Bell Labs. Since then, the FG device has been extensively developed from the aspects of principles, structures, and materials, demonstrating broad applicability in areas such as memory, synaptic electronics, and neuromorphic computing. Fig. 4 detailedly illustrates the evolution of FG devices in terms of the timeline of milestones. FG transistors commonly consist of a control gate, blocking layer, FG, tunneling layer, semiconductor channel, and source/drain electrodes (Fig. 5A). Compared to conventional MOSFETs, the FG structure incorporates an additional FG layer and a tunnel layer, allowing the FG transistor to store information even when power is disconnected. The storage mechanism operates by capturing and releasing carriers within the FG layer. By applying a specific voltage to the gate of the FG transistor, charges can be injected into or removed from the FG (known as program and erase process). Driven by the dielectric layer, the capture and release of charges are conducted by the FG layer, ensuring that captured charges remain even without any external electric field, thus achieving nonvolatile behaviors [34].

The capture and release process of carriers in FG involves several mechanisms: channel hot electron injection [119–124], Fowler–Nordheim tunneling [125–128], and direct tunneling [129–133]. In channel hot electron injection, hot electrons refer to a subset of electrons that gain high energy due to acceleration by a strong transverse electric field (Fig. 5B). During programming process, high voltages are applied between the source–drain (*V*_ds_) and gate (*V*_g_), creating strong transverse and vertical electric fields. The current flows from the source to the drain, and electrons near the drain end of the channel are accelerated by the transverse electric field. Once the electrons gain enough energy, they will overcome the barrier potential and be captured by the FG under the assistance of the vertical electric field [134]. The change in the floating gate’s charge state alters the transistor’s threshold voltage, enabling information storage. This mechanism requires the coordinated action of high *V*_g_ and *V*_ds_ but allows dynamic control of stored information through voltage adjustments. To achieve efficient injection and stability, it is necessary to optimize the device structure (such as the drain-end electric field distribution) and barrier layer materials (such as thickness and dielectric properties) to balance injection efficiency and reliability [135].

Fowler–Nordheim tunneling is a quantum tunneling effect that occurs when electrons pass through a thin barrier under a high electric field (Fig. 5C). This mechanism was first proposed by Fowler and Nordheim [136] in 1928 to describe the tunneling of electrons through the barrier between metal and vacuum under a strong electric field. In semiconductor devices, when a high voltage is applied, a strong electric field is formed within the insulating layer, narrowing the effective width of the barrier. Even if the electron energy is below the height of the potential barrier, there is still a certain probability of tunneling to the other side [137]. When a sufficient number of electrons tunnel through the barrier, a measurable current is formed, which depends on both the applied electric field strength and the properties of the barrier. Since the current density exhibits an exponential dependence on the electric field strength, compared to hot electron injection, Fowler–Nordheim tunneling consumes less overall power due to the extremely low current, but the high voltage required may cause degradation of the insulating layer and affect long-term reliability. In practice, there is a trade-off between barrier height, insulating layer thickness, and electric field distribution to optimize tunneling efficiency and device lifetime [138].

Direct tunneling refers to the process where electrons directly tunnel through a thin barrier without any energy excitation (Fig. 5D). This effect usually occurs in very thin tunneling layers. This process is characterized by faster programming speeds, and the tunneling current is highly dependent on the barrier thickness. Thinner barriers allow for larger tunneling currents and, thus, faster programming at low voltages. However, too thin a barrier leads to a higher risk of charge leakage or oxide breakdown, and performance and reliability need to be balanced by optimizing materials such as high-κ dielectrics. Unlike Fowler–Nordheim tunneling, direct tunneling does not require a strong electric field to tilt the barrier and is suitable for low-power nanodevices.

#### Structural classification of 2D FG transistors

**Fig. 6.**
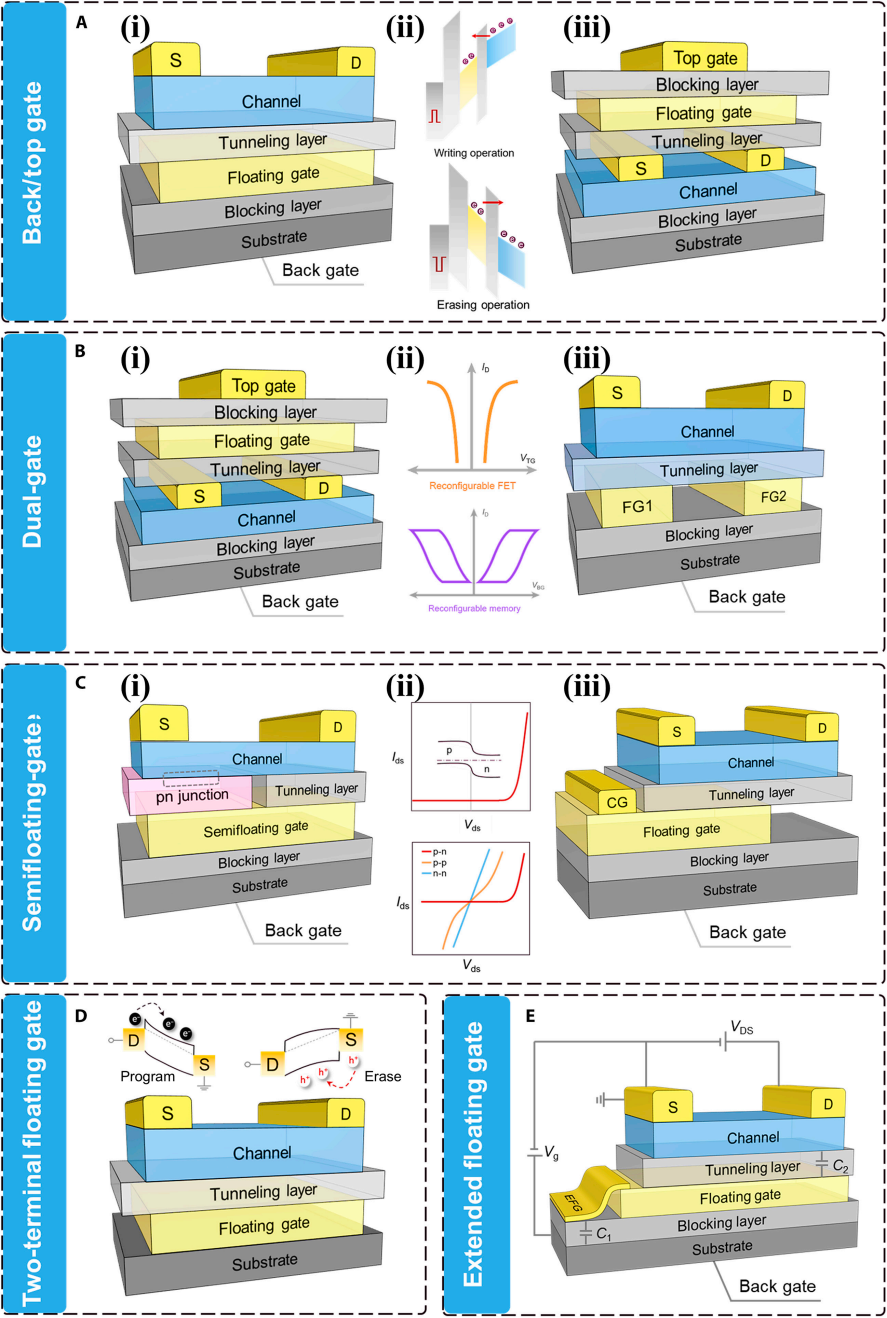
Typical structure of FG device [139]. (A) Typical structure of back gate and top gate FG device and write/erase operation. (B) Typical structure of dual-gate FG device and its reconfigurable functions. (C) Typical structure of semifloating gate device and characterization of reconfigurable homojunction. (D) Typical structure of two terminal FG device and write/erase operation. (E) Typical structure of extended FG device. EFG, extended floating gate; CG, control gate.

The fundamental operation principle of FG transistors involves using the charge in the FG to control the channel’s conductivity and modulating charge carrier density. Adjusting the voltage applied on control gate allows charge capture/release within the FG, enabling information storage and retrieval [134]. In current research, 2D materials have been extensively used to construct FG transistors. Their atomic thickness helps to efficiently reduce energy dissipation induced by short-channel effects [13–15]. On the basis of different stacking methods of 2D materials, device performance, charge storage, specific applications, and working principles in current research, 5 typical FG structures have been identified [139]: back/top gate, dual gate, semifloating gate (SFG), 2-terminal floating gate (2TFG), and extended floating gate, as shown in Fig. 6.

• Back/top gate. Single-gate control is the most commonly used modulation strategy in FG devices, which can be categorized into back gate and top gate based on the gate position. For the back gate configuration, as shown in Fig. 6A-i, the control gate is located below the FG layer and the gate insulating layer. In silicon-based FG devices, the bottom gate is usually formed by growing an insulating oxide layer (such as SiO_2_) on the silicon substrate, making its fabrication process relatively simple. In FG transistors that utilize 2D channel layer, adjusting the applied gate voltage enables effective control over the type and concentration of charge carriers in the channel, as shown in Fig. 6A-ii, allowing for precise modulation of the device’s electrical performance. For instance, Liu et al. [91] manufactured a ultrafast nonvolatile flash memory using a MoS_2_/h-BN/multilayer graphene vdW heterostructure bottom-gate transistor, achieving ultrafast write/erase speeds of 20 ns, on/off current ratio of memory state at ~10^6^, and nonvolatile retention properties. In the top-gate structure, as illustrated in Fig. 6A-iii, the control gate is located above the FG layer and the gate insulating layer. Under the same dielectric layer material (e.g., SiO₂), the top-gate structure can optimize the carrier transport efficiency due to the enhanced electrostatic control at the gate–channel interface, which improves the subthreshold swing (*SS*) and threshold voltage stability. However, the absolute value of the operating voltage is still largely dependent on the dielectric layer thickness and dielectric constant. The advantages of the top-gate design need to be evaluated in conjunction with the device structure and material properties. Chen et al. [86] designed a top-gate transistor based on an h-BN/graphene/h-BN/MoS_2_ structure, which can be utilized for simulating human memory systems. This system integrates 3 types of memories, including sensory memory (~milliseconds), short-term memory (~seconds), and long-term memory (~kiloseconds). The device operates at lower voltages, demonstrating a range of functions based on the memory system, such as forgetting, memory transition, memory level potentials, and inhibition. In addition, the designed human memory system is capable of logical decision-making operation and can store the decision results in situ.
• Dual gate. Dual-gate FG devices feature 2 common structures. In the structure depicted in Fig. 6B-i, the FG device comprises 2 control gates (top/bottom gate). Compared to single-gate control, this configuration allows for more flexible electronic performance tuning, achieving larger on/off current ratios and lower energy dissipation. The 2 control gates can independently modulate channel carriers from both sides of the device, providing finer electric field control to reduce the threshold voltage and static power consumption. Pang et al. [140] designed a dual-gate FG transistor with a MoS_2_ channel, featuring a bottom gate and an In_2_Se_3_ top gate. The independent dual-gate control affords the device a high on/off current ratio of 10^6^ and a low subthreshold swing of 94.3 mV·decade^−1^, along with logic functionalities. The dual-gate coupling effect turns the top gate into a charge trapping layer, thereby enabling nonvolatile memory behavior (on/off ratio of ~10^5^ and retention time of >10^4^ s) and 6-level memory states. Moreover, because of the interface coupling between the dual gates, the device exhibits tunable photoelectric detection functionality, with a response rate of ~857 A·W^−1^, along with photomemory characteristics. The dual-gate structure paves the way for combining sensation, logic operation, and data storage into a single device. In the dual-gate structure as shown in Fig. 6B-iii, it divides the traditional bottom gate into 2 split gates. Sun et al. [93] reported a split-gate FET based on graphene/h-BN/WSe_2_, capable of acting as a reconfigurable transistor and nonvolatile memory to provide logical functionalities, as illustrated in Fig. 5B-ii.
• SFG. In 2013, Wang et al. [141] first introduced the concept of SFG transistors, ingeniously integrating a tunnel FET with FG devices to create a new type of “SFG” structure. The SFG is a hybrid structure that lies between traditional floating gates and fully depleted transistors. It is characterized by charge storage achieved through capacitive coupling between the partially floating gate and the channel. Unlike traditional floating gates, the charge storage region of the SFG is isolated from the channel by a dielectric layer, while still retaining direct electrostatic control. SFGs enable charge injection/erasure with small voltage changes due to capacitive coupling effects. At the same time, the partially isolated design reduces charge leakage paths, which contributes to improved data retention. This structure offered a compromise between nonvolatile storage and rapid switching operations, characterized by its small size, low power consumption, simple fabrication process, and faster data writing/erasing speeds, all achievable at low-voltage conditions. However, the charge storage density of SFG is usually lower than that of conventional floating gate, and the fabrication process is more complex, requiring precise control of the dielectric layer thickness and interface quality. Liu et al. [142] showcased a nonvolatile SFG memory device based on a stacked 2D vdW heterostructure, as depicted in Fig. 6C-i. This device exhibited refresh interval 156 times longer than dynamic random access memory, reduced power consumption by frequent refresh operations, and ultrafast writing operations on nanosecond scale. The SFG structure substantially improved the writing process, achieving 10^6^ times faster than that of other 2D-material-based memories. Another type of SFG structure, as shown in Fig. 6C-iii, could implement multiple programmable nonvolatile functionalities of p-n and n^+^-n junctions [30]. Wang et al. [94] constructed a multifunctional SFG transistor based on an InSe/h-BN/multilayer graphene vdW heterostructure, which is capable of electrostatic programming within nanoseconds (20 ns). By applying electrical pulses in different directions, different types of homojunctions (e.g., lateral p-n and n^+^-n) could be formed, modified, and reversed as shown in Fig. 6C-ii. The p-n homojunction achieved a high rectification ratio of about 10^5^, allowing dynamic switching between 4 different conduction states across 9 orders of magnitude in current, enabling its application as a logic rectifier, memory, and multivalued logic inverter.
• 2T floating gate. The 2TFG devices were first proposed by Vu et al. [84] (Fig. 6D), which featured a design of vertically stacked single-layer MoS_2_/h-BN/single-layer graphene. When the h-BN layer has a proper thickness, charges can readily tunnel through the h-BN layer to FG due to the large potential difference. In contrast, charges stored in the graphene remain trapped in the FG, as the absence of a potential difference prevents their tunneling to the source [84]. This 2TFG structure also offers advantages in photomemory, exhibiting large on/off current ratio and long retention time [143–148]. However, the application of these photomemory devices is hindered by the high off-state current/programming voltage/energy dissipation. These limitations are partly due to the reliance on a 3-terminal structure involving gate, source, and drain, which complicates the miniaturization of the devices, as well as scalability and the integration of functionalities into circuits. To overcome these challenges, Tran et al. [88] designed a multilevel nonvolatile photomemory device utilizing a 2TFG FET with a MoS_2_/h-BN/graphene heterostructure. This device achieved an ultralow off-state current of about 10^−14^ A and a superhigh optical on/off current ratio of ~10^6^, supporting 18 different current levels, equivalent to more than 4 bits of information storage. Moreover, it showcased exceptional durability with over 10^4^ program/erase cycles and an extended retention time of over 3.6 × 10^4^ s, with a programming voltage as low as −10 V. This exhibits an important step toward the miniaturization and high-density integration of photomemory devices using vdW heterostructures, providing a potential direction to future developments in this area.
• Extended floating gate. Because of the challenge of high power consumption in traditional FG devices with large operating voltage, Gupta et al. [149] introduced an expanded FG structure (Fig. 6E). They ingeniously connected the bottom layer of the graphene FG to a floating metal electrode by photolithography. This connection increased the total capacitive area between the FG layer and the bottom Si^++^ gate (*C*_1_), which results in *C*_1_ ≫ *C*_2_ (FG − channel capacitance) and effectively enlarges the effective gate capacitance. The increased effective capacitive area results in an increase in the effective gate voltage [150], thereby enhancing the control gate/channel coupling. This improvement in device performance resulted in remarkably low subthreshold swing (77 to 80 mV/decade) and ultralow power consumption [20 pJ for long-term depression (LTD) and <0.3 pJ for long-term plasticity (LTP)]. This innovative approach effectively tackles the critical challenge to developing more energy-efficient electronic components. The extended floating gate increases the coupling capacitance by enlarging its area, thereby enhancing charge storage capability. However, the expansion of its physical dimensions inherently conflicts with the trend of miniaturization in integrated circuits. This is manifested in inefficient area utilization (with storage density decreasing as the area increases, limiting high-density integration) and an increase in parasitic capacitance (the additional capacitance introduced by the extended structure may reduce read/write speeds and exacerbate crosstalk noise). FG transistors that integrate 2D materials showcase remarkable potential for applications in nonvolatile memory, artificial synapses, and neuromorphic computing owning to their unique physical properties and structural advantages. Hereinafter, we summarize the current state of research in this area and explore the future prospects of FG transistors that incorporate 2D materials.

## Applications of FG Devices Based on 2D Materials

### Nonvolatile memory

FG transistor structure includes an extra FG layer along with a tunneling layer, enabling it to retain data even when powered off. This memory function operates by capturing and releasing charge carriers within the FG layer. When a specific gate voltage is applied to the device, carriers can either be injected into or extracted from the FG layer, allowing for programmable charge storage. Charge carrier transport can be regulated through various types of stimuli, typically categorized into electrical, optical, and mechanical methods. Each of these approaches represents distinct memory mechanisms, which are outlined in detail below.

**Fig. 7.**
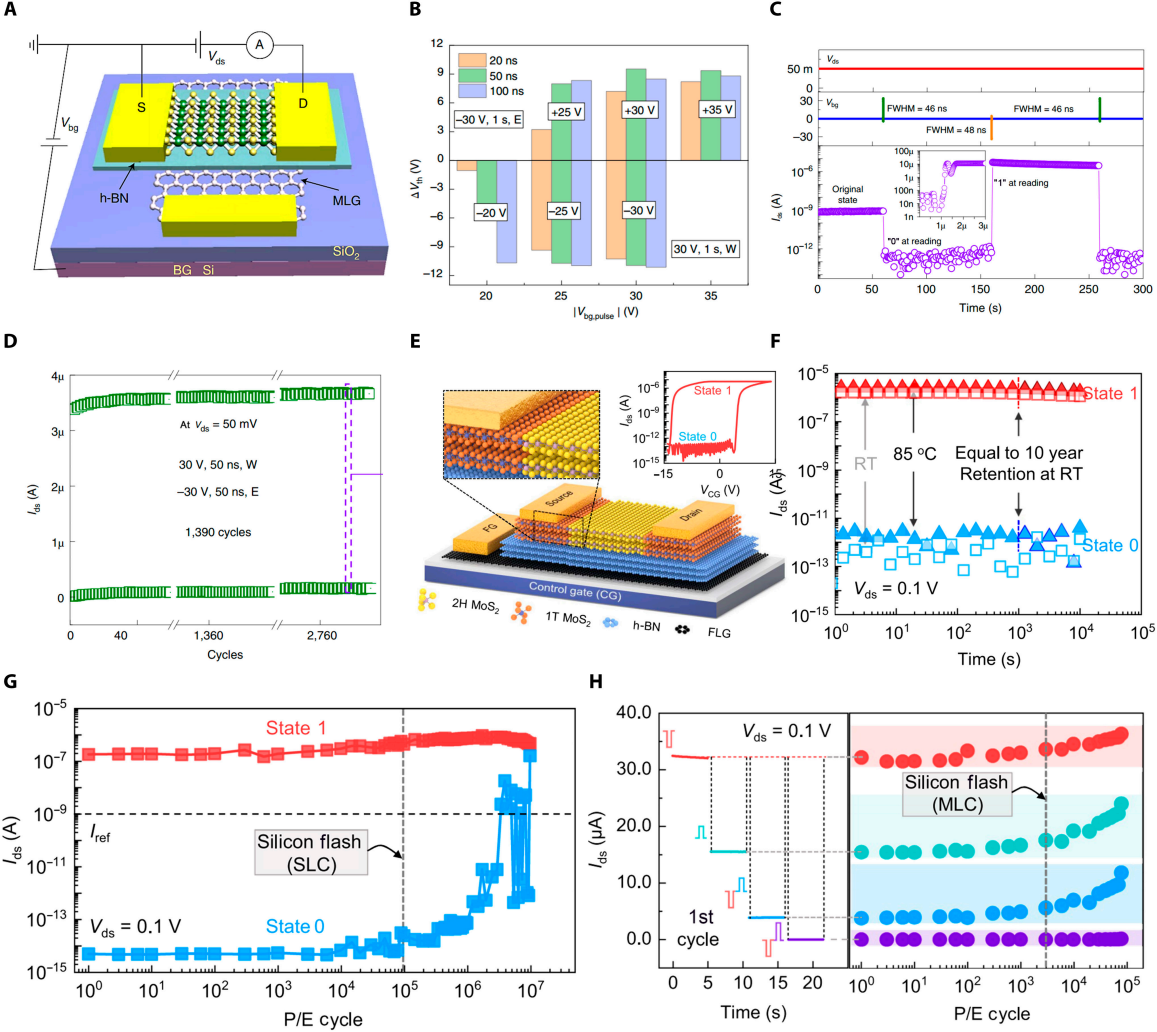
Electrical-modulation-based nonvolatile FG memory device. (A) Device structure of vdW-heterostructure-based ultrafast flash memory utilizing MoS_2_/h-BN/multilayer graphene. (B) Illustrations of the threshold voltage applying different pulses. (C) Ultrafast writing/erase operation (50 ns). FWHM, full width at half maximum. (D) Endurance performance of the FG memory device [91]. (E) Illustration of MoS_2_/h-BN/few-layer graphene vdW heterostructure; the edge contact is created by patterning 1T-LixMoS_2_ placed under a conventional Cr metal contact. (F) Retention performance of the FG memory under different temperatures. RT, room temperature. (G and H) Endurance characteristics of the FG memory as single-level cells (SLCs) (G) and multilevel cells (MLCs) (H) [95]. P/E cycle, program/erase cycle.

**Fig. 8.**
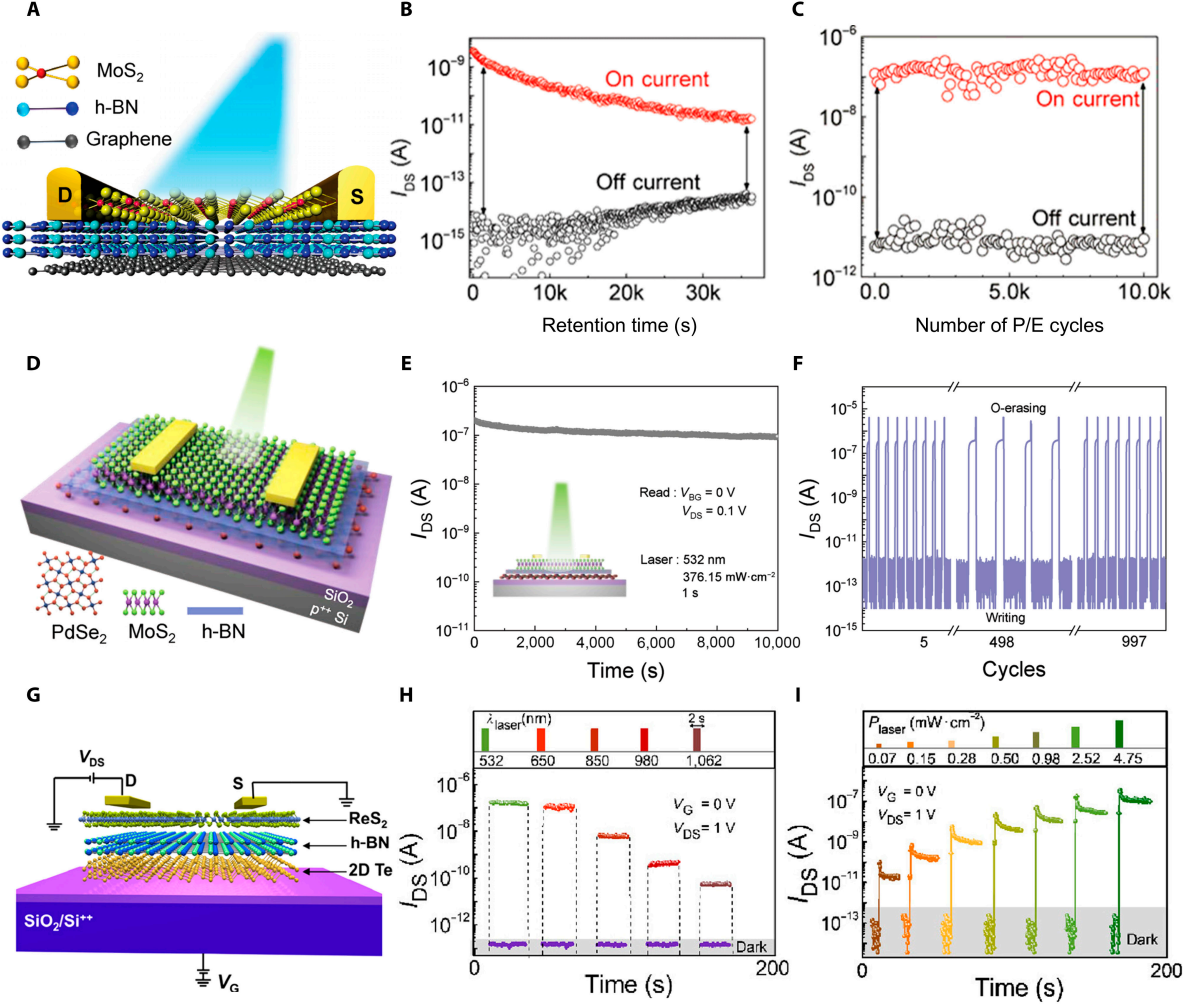
Optical modulation based nonvolatile FG memory device. (A) An optical memory device utilizing an FG FET with a MoS_2_/h-BN/Gr heterostructure. (B and C) Retention (B) and endurance (C) performance of the FG device in off and on current [88]. (D) Schematic representation of the MoS_2_/h-BN/PdSe_2_ optoelectronic nonvolatile FG transistor. (E) Retention behavior of the device following laser irradiation. (F) Endurance properties of writing and optical erasing [144]. (G) Schematic representation of the ReS_2_/h-BN/2D Te heterostructure FG memory device. (H) Laser-mediated multibit storage in the ReS_2_/h-BN/2D Te vdW FG transistor, with electrical writing conducted at various laser pulse wavelengths and a power intensity of 4.756 mW/cm^2^. (I) Electrical programming at various laser pulse power levels, *t*_pulse_ = 2 s at *V*_G_ = 0 V and *V*_DS_ = 1 V [143].

**Fig. 9.**
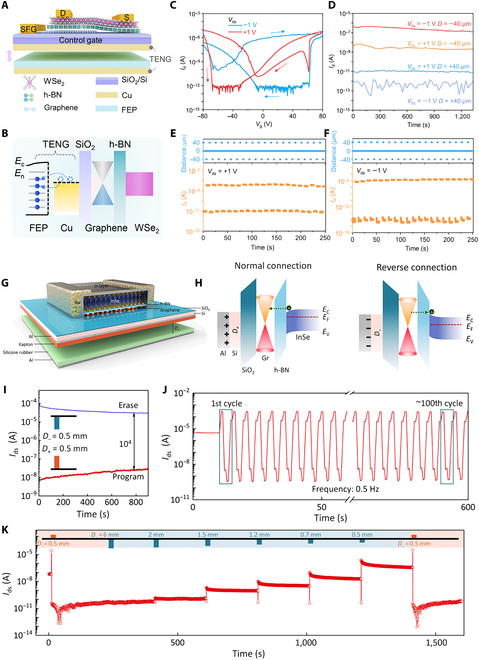
Mechanical-modulation-based nonvolatile FG memory device. (A) Illustration of the structural design of a Gr/h-BN/WSe_2_ SFG synaptic transistor with a TENG. (B) Schematic representations of the operating mechanism and the corresponding energy band diagram in the initial state. (C) Transfer curves of the device measured at *V*_ds_ = ±1 V. (D) Retention characteristics under different junction states achieved by applying varied *D* pulses at *V*_ds_ = ±1 V. (E and F) Endurance performances of the device for 20 cycles of the device, showcasing switching behaviors between writing and erasing states under periodic alternating displacements [172]. (G) Illustration of the InSe/h-BN/Gr heterostructure FG device with a TENG. (H) Band diagrams of the InSe triboelectric under positive/negative *V*_gs_ coupled different separation distance *D*_-_/*D*_+_. (I) Retention characteristics following programming and erasing potentials with 0.5 s at *V*_ds_ = 0.5 V. (J) Endurance performance test evaluated about 100 programming/erasing cycles with a distance pulse of −0.7 mm/+1.5 mm for 2 s. The read gate voltage *V*_read_ at 0 V with the drain voltage set to *V*_ds_ at 0.5 V. (K) Multilevel data storage characteristics of the memory device evaluated at different TENG separation distances, with *V*_ds_ = 0.5 V [25].

• Electrical modulation. Extensive research has been conducted on electrically controlled FG devices for flash memory applications, where applying bias to the gate effectively controls the charge transport in the FG, offering solutions to fast, high-density, and reliable storage. Since the invention of FG memory in 1967, flash memory has been widely adopted in nonvolatile memory applications. However, its relatively low programing speed has limited the use of flash memory in high-speed on-chip memory applications. A larger gate coupling ratio (GCR) and smaller tunneling barriers would enhance programming efficiency. The large electron/hole energy barriers of Si/SiO_2_ are the main limitations on the programming speed of silicon-based memories. The use of 2D heterostructures would greatly enhance the programming efficiency. Thus, Liu et al. [91] fabricated a vdW-heterostructure-based ultrafast flash memory utilizing MoS_2_/h-BN/multilayer graphene (Fig. 7A). Leveraging a rational GCR, small carrier tunneling barriers, and atomically flat interfaces, they showcased a nonvolatile flash memory with ultrafast writing/erasing speed. This FG device maintains conventional flash memory’s advantages of nonvolatility and high-density while achieving writing speeds comparable to volatile dynamic random access memory. Bilayer MoS_2_ was used as channel, multilayer graphene as the FG layer, and 10.5-nm-thick h-BN as the tunneling layer. By quantifying the width and magnitude of writing/erasing pulses, the memory was identified to have multilevel data storage capacity (Fig. 7B). Under ultrafast *V*_bg_ transient current responses, the memory demonstrated a writing/erasing ratio of >10^6^ (Fig. 7C). Through dynamic monitoring of the device, the memory exhibited stable nonvolatile characteristics (Fig. 7D). In addition to controlling an appropriate GCR and tunneling barriers, ideal Schottky contacts are considered to have the capability of injecting hot carriers into the channel under applied gate and have the potential to greatly enhance the flash memory’s performance [151]. However, achieving abrupt Schottky contacts in traditional silicon-based technology is challenging because of interface diffusion and Fermi level pinning effects [151,152]. However, 2D materials, with their atomically flat layered structure, allow for the design of ideal Schottky barriers in many ways, including phase engineering and vdW contacts [153,154]. They can also be prepared in high densities with low-defect interfaces, making them ideal for providing optimal charge injection interfaces and durable tunneling layers for ultrafast and robust storage operations [155,156]. Yu et al. [95] demonstrated a 2D MoS_2_ flash memory based on phase-transition edge contact (Fig. 7E), utilizing chemical Li^+^ intercalation to transform MoS_2_ within the traditional metal–semiconductor contact region from the semiconducting phase (2H) to the metallic phase (1T), transitioning the device’s metal–semiconductor contact type from traditional 3D/2D surface contact to 2D/2D type edge contact with an atomically sharp interface. This design enabled ultrafast programming/erasing speeds (~10/100 ns) and excellent stability (endurance over 10^6^ cycles), with a superior on/off ratio exceeding 10^7^ from 10-ns programming and 100-ns erasing, and an equivalent data retention life span of over 10 years at room temperature (Fig. 7F). Moreover, the memory operates at significantly low voltages due to its excellent charge injection efficiency, providing an exceptional endurance of over 3 × 10^6^ cycles (Fig. 7G) and multilevel storage capacity (Fig. 7H). This structure markedly improved the efficiency of charge injection into the FG and realized ultrafast and ultrarobust memory operations.
• Optical modulation. Optically controlled FG transistors, using light signals to modulate charges on the FG, introduce a new control mechanism with a series of unique advantages. First, noncontact control through light signals allows for the manipulation of device functions without physical contact, reducing wear during operation and enhancing device reliability and lifespan. Utilizing light sources of different wavelengths enables selective activation of specific areas within the FG transistor, increasing design flexibility. By integrating optoelectronic functions, optically controlled FG devices combine photodetection and charge storage capabilities, applicable in fields such as photodetection and image sensing [157]. Currently, mainstream photonic memory devices rely on a 3-terminal transistor structure, limiting device miniaturization and adding complexity to scalability and integration of functions. To overcome this problem, Tran et al. [88] fabricated a 2TFG photonic storage device based on a MoS_2_/h-BN/graphene heterostructure (Fig. 8A), which is programmed by applying negative source–drain voltage and erased by light pulse irradiation. The initial programming/erasing current ratio reached 10^6^, with a prolonged retention time (Fig. 8B). After 10^4^ cycles of writing/erasing, both the on current and off current remained essentially unchanged, indicating high durability and stability of the device (Fig. 8C). Current research has largely focused on FG structures based on multilayer graphene. Compared to graphene, TMDCs offer superior photoresponse, delivering more opportunities to achieve optoelectronic FG memory devices by integrating TMDCs into FG. For instance, Wang et al. [144] showcased an optoelectronic FG memory with a MoS_2_/h-BN/PdSe_2_ structure (Fig. 8D), where MoS_2_, h-BN, and PdSe_2_ sheets serve as the semiconductor channel, tunneling layer, and FG layer, respectively. This device achieves optical erasure under a 530-nm laser pulse within 2.3 ms, reaching a 5-order magnitude difference between photoinduced writing and erasing, demonstrating superior optoelectronic performance. This work surpasses previous reports on 2D heterostructure optoelectronic memory devices in terms of high-speed optical erasure. The device also features reliable retention characteristics; when a laser pulse is applied, the erased state current maintains at the level of 10^−7^ A without any decay after 10^4^ s (Fig. 8E). Durability tests show stable performance within 10^3^ cycles of electrical writing/optical erasure, indicating robust and repeatable properties (Fig. 8F). Considering the demands for multifunctional memory devices, nonvolatile FG devices must exhibit high on/off ratios, wide absorption spectra, long-term data retention, and robust durability [158,159]. Bach et al. [143] conducted a systematic study and demonstrated a high-performance optical memory device based on a ReS_2_/h-BN/2D Te vdW heterostructure (Fig. 8G). Because of the exceptional optoelectronic characteristics of ReS_2_ and 2D Te, the memory device exhibited remarkable long-term stability, high on/off current ratio, and broad spectral response capability. The narrow bandgap of 2D Te endowed the device with wide-band optical programmability at room temperature (visible to near-infrared regions). By applying laser pulses in different wavelengths, various current levels were achieved, showcasing the memory’s capability for multilevel data storage across a wide optical writing range (Fig. 8H). Furthermore, by controlling power density with a fixed laser wavelength, different current levels were adjusted, realizing more complex data patterns and larger data volume. Even when the light pulses in small intensity are applied in step, the memory can achieve more than 7 states (Fig. 8I).
• Mechanical modulation. In 2012, Wang and colleagues [160,161] introduced the triboelectric nanogenerator (TENG) based on the ubiquitous contact electrification effect, offering a universal method for converting different forms of mechanical energy from the environment into electrical energy as micro/nanopower sources. Coupling TENG with semiconductor devices allows the conversion of mechanical energy into electrical potential under mechanical stimuli, modulating the carrier transport properties and enabling active and direct coupling between electronic devices and external environment [162–170]. This control mechanism is termed as tribotronics [171]. Jia et al. [172] demonstrated a reconfigurable nonvolatile device integrating TENG-driven triboelectric potential gate-controlled WSe_2_/h-BN/graphene SFG FET heterostructure stack (Fig. 9A). The TENG consists of Cu/FEP (fluorinated ethylene propylene)/Cu, where one fixed Cu friction layer is attached to the transistor’s gate and the other side is a movable friction layer (FEP/Cu). As shown in Fig. 9B, the tribopotential can adjust the device’s band structure, modulate the charge transfer in FG layer, and complete programming/erasing process by inducing positive or negative potentials equivalent to applying positive or negative voltages to transistor gate. Unlike conventional FG transistors, the SFG device exhibited 2 different hysteresis windows, excellent reliability with an on/off current ratio of 10^6^ (Fig. 9C) and a retention time of 1,200 s at *V*_ds_ = −1 V (Fig. 9D). The device can stably implement “on” and “off” state transitions under different source–drain bias conditions through TENG-regulated writing/erasing dynamic cycles (Fig. 9E and F). Gao et al. [25] reported a touch modulated FG transistor by using TENG integrated with InSe/h-BN/Gr vdW heterostructure (Fig. 9G). This device was manufactured using a simple copper grid mask technique without electron beam lithography commonly used in most 2D FG device fabrication and successfully achieved high mobility and low energy dissipation. The device’s band structure was adjusted by varying the separation distance between the 2 friction layers (Fig. 9H), which was realized by coupling different static potentials of TENG to the transistor gate. This process achieved a nonvolatile memory with an on/off ratio of about 10^4^ (Fig. 9I), excellent repeatability over more than 100 cycles (Fig. 9J), and multilevel dynamic storage (Fig. 9K).

### Artificial synapses for neuromorphic systems

Three-terminal FET devices can be readily utilized to simulate synapses (known as synaptic transistors or neuromorphic transistors), exhibiting tremendous potential in artificial neural networks (ANNs) and neuromorphic computing [173–182]. In biological neural networks, synapses are structures that connect 2 neurons and transmit signals. Signals are transmitted from one neuron (presynaptic neuron) to another (postsynaptic neuron) through neurotransmitters. In 3-terminal synaptic transistors, this process is emulated as follows: The gate is used to simulate the presynaptic neuron, receiving signals from the external environment; the source and drain simulate the postsynaptic neuron, where the source–drain current flowing models the signal transmission process; the carriers in the channel are regarded as neurotransmitters, with the gate voltage controlling the channel conductivity to simulate changes in synaptic strength [183–186]. These 3-terminal synaptic devices can simultaneously capture and recognize stimuli, featuring highly stable and well-controlled electrical performance [187–190]. Significantly, the surface carrier transport in 2D materials is easily influenced by the surrounding environment (e.g., electric fields and dipoles) [191–193], providing a platform for developing high-performance synaptic transistors. Furthermore, FG transistors incorporating 2D materials have prominent advantages in realizing certain types of synaptic behaviors and functions due to the nonvolatile nature, especially LTP and LTD involving long-term information storage and learning capabilities of neural synapses [194,195]. This allows the updates in synaptic weights to remain unchanged without a continuous power supply, simulating the biological neural synapses’ ability to make long-term adjustments in connection strength after a series of activations [196–198]. This is crucial for designing neuromorphic computing systems capable of emulating complex learning and memory mechanisms. Corresponding modulation methods can be categorized into electrical, optical, and mechanical modulation.

**Fig. 10.**
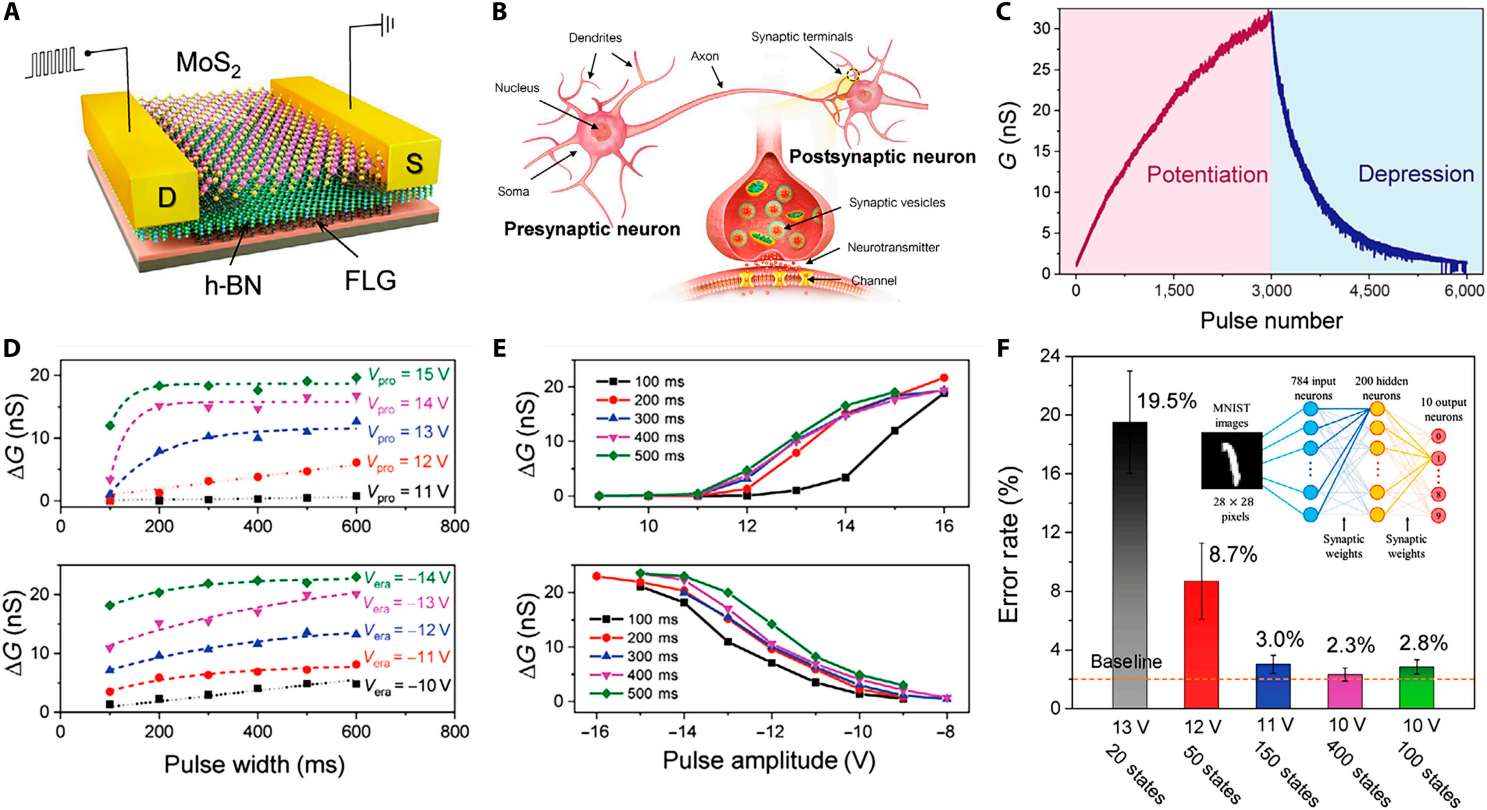
Electrical-modulation-based artificial synapse FG device. (A) Schematic illustration of the all-2D-materials 2TFG memory (2TFGM). (B) Schematic illustration of biological neurons, highlighting the soma, axon, dendrites, and synapses, which serve as the connections between adjacent neurons. The enlarged area depicts the synaptic transmission process. (C) Postsynaptic current as a function of pulse number, illustrating the LTP and depression behaviors replicated by the 2TFGM device. (D and E) Device conductance variations as a function of pulse width (D) and amplitude (E) during the P/D process. (F) Error rate after 20 training epochs with different P/D processes [20].

**Fig. 11.**
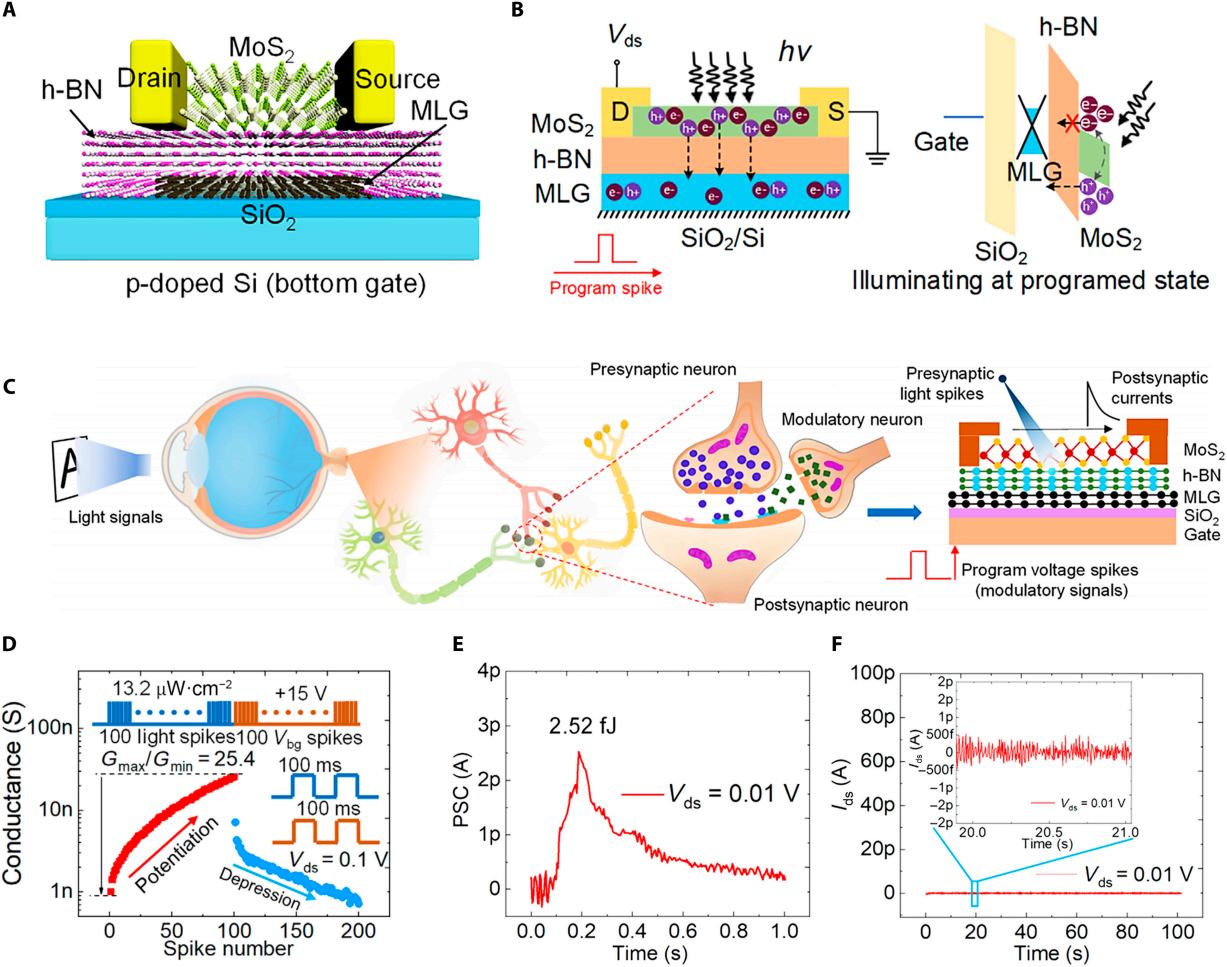
Optical-modulation-based artificial synapse FG device. (A) Schematic representation of an optoelectronic synaptic FG transistor utilizing multilayer graphene/h-BN/MoS_2_ vdW heterostructures. (B) Illustration of light-induced carriers tunneling mechanism, depicted using energy-band diagrams. (C) Schematic diagram of biological retina and synapses, alongside a comparative analogy with FG transistors. (D) Photonic potentiation and electric depression behavior of FG device. (E) The postsynaptic current was induced by a single light pulse (470 nm, 100 ms, and 13.5 μW/cm^2^) applied under a *V*_ds_ of 0.01 V. (F) The *I*_ds_ was measured in the dark under a *V*_ds_ of 0.01 V in the dark for 100 s and recorded as the noise current [24].

**Fig. 12.**
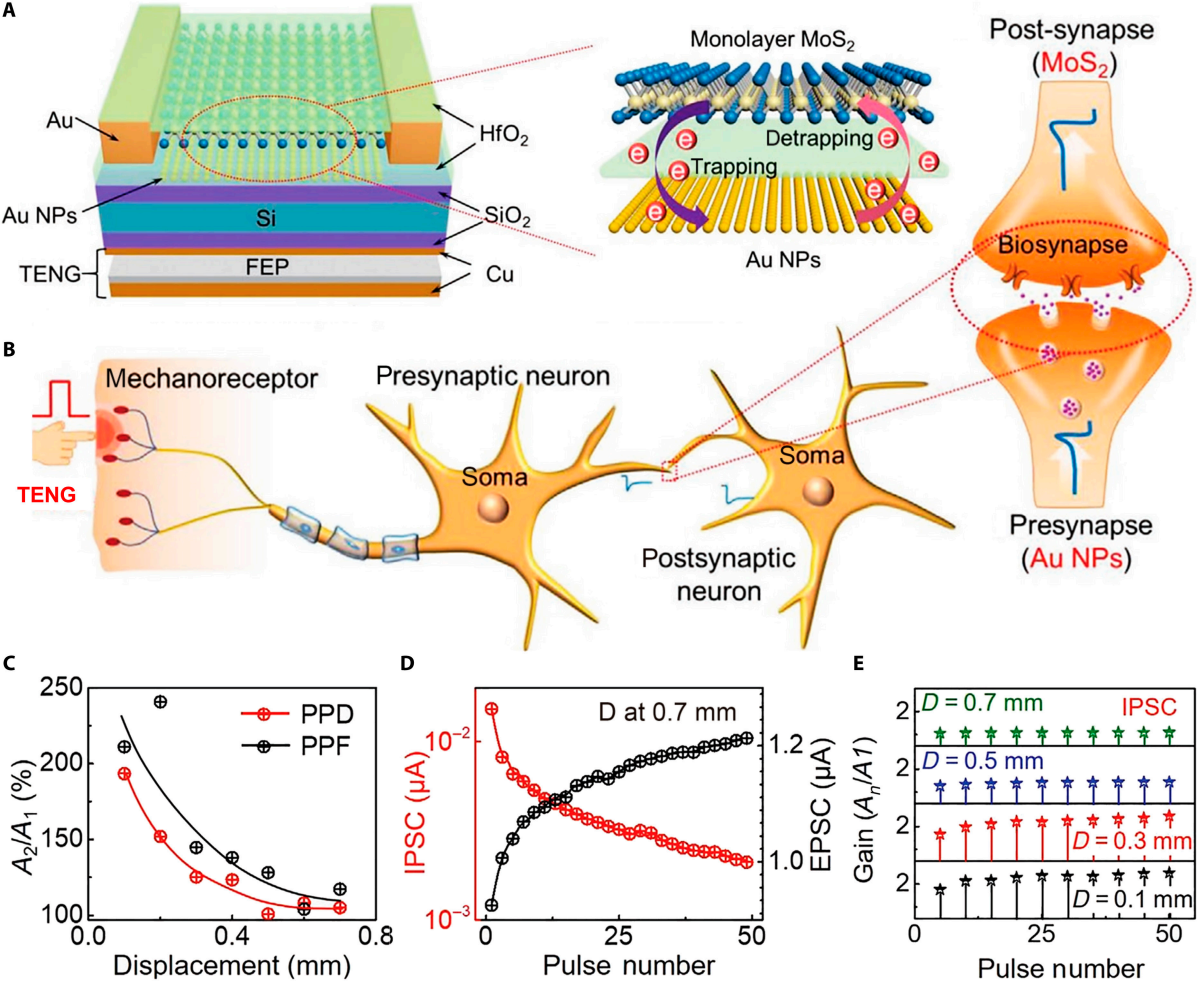
Mechanical-modulation-based artificial synapse FG device. (A) Diagrammatic representation of the mechanoplastic MoS_2_ synaptic transistor (left), accompanied by an explanation of the operational mechanism of the Au-nanoparticle-based FG layer (right). (B) Comparison between a biological nervous system and the presented device, with an enlarged view illustrating the functioning of a biosynapse and its analog to the trapping/detrapping process in the device. (C) Displacement-dependent PPD/F index (*A*_2_/*A*_1_) of the mechanoplastic FG synaptic transistor. (D) IPSC and EPSC of the synaptic transistor with *D* pulse at 0.7 mm. (E) Depression gain (*A_n_*/*A*_1_) as a function of pulse number with applied *D* pulses at distance of 0.1, 0.3, 0.5, and 0.7 mm [27].

• Electrical modulation. Electrically controlled artificial synaptic devices have been extensively reported [199–210], but issues such as reliability, energy consumption, linear/symmetric weight updates, and the limited number of states remain major barriers for their practical implementation in high-energy-efficiency neuromorphic computation. To address these problems, Tang et al. [20] designed all-2D-materials-based FG memories (2D-FGMs) as artificial synapses (Fig. 10A), achieving highly linear/symmetric weight updates with low energy dissipation and high reliability. The structure of biological neurons is shown in Fig. 10B, and by adjusting the conductance states in the 2D-FGM device, 2 crucial synaptic functions (LTP and LTD; Fig. 10C) can be readily emulated, indicating that the device has lots of states available for neuromorphic computing. It is noteworthy that the linearity, on/off ratio, and the number of analog states in the 2D-FGM artificial synapse can be adjusted by controlling the amplitude/width of the applied pulses (Fig. 10D and E). Image classification relying on experimental potentiation/depression (P/D) results in different linearities can be implemented assisted with the MNIST dataset for simulations, which can achieve the recognition accuracy as high as 97.7% (Fig. 10F). The 2D-FGM is one of the ideal options for building energy-efficient neuromorphic systems.
• Optical modulation. Recently, optoelectronic neuromorphic devices that integrate nonvolatile memory and optical interaction similar to biological synapses have demonstrated great advantages in artificial vision systems and neuromorphic computing due to their higher information processing capability and lower energy consumption. These artificial optoelectronic synaptic devices emulate synaptic behaviors under light stimulation based on the generation of nonvolatile photocurrents [211–213]. However, most studies attribute the source of nonvolatile photocurrents to the capture/release process of photogenerated carriers by local or interfacial defects, remarkably hindering the development of reliable and efficient platform for neuromorphic hardware [214]. To address this, Sun et al. [24] designed an FG photonic synaptic transistor with graphene/h-BN/MoS_2_ vertical vdW heterostructure (Fig. 11A), integrating nonvolatile memory and photosensitive properties for neuromorphic photonic applications. Through the electrical and optical comodulation on the vdW heterostructure for photogenerated carrier tunneling, multiple synaptic functions, such as short-term/long-term plasticity, and paired pulse facilitation were successfully emulated. Fig. 11B shows the dynamic response of photogenerated carriers under FG storage operations and the band alignment of the heterostructure. Compared with biological synapses, the photonic FG synaptic transistor (Fig. 11C) uses the light spike as a presynaptic stimulus and the photocurrent as the postsynaptic current. Simultaneously, the applied *V*_bg_ is regarded as an external modulation signal, simulating the regulatory function of synapses in complex neural systems. This voltage control enables the manipulation of synaptic plasticity. Photon-enhanced and electrically suppressed behaviors were obtained by applying light spikes and *V*_bg_ spikes (Fig. 11D), where *G*_max_/*G*_min_ = 25.4 (>10) was achieved for efficient neural networks and beneficial for high-precision recognition in artificial vision systems. Moreover, the device exhibits ultralow power consumption, requiring only ~2.52 fJ per light spike at *V*_ds_ = 0.01 V (Fig. 11E), demonstrating that synaptic functions can be achieved at ultralow operating voltages (*V*_ds_ = 0.01 V and *V*_gs_ = 0 V) due to the ultralow noise current in the programming state (Fig. 11F). The fabricated optoelectronic synaptic transistor shows remarkable promise for neuromorphic computing and sensing applications. It overcomes the drawbacks of traditional artificial synapse, e.g., narrow bandwidth, low speed, high cross-talk, and limited functionality.
• Mechanical modulation. Triboelectric-potential-modulated synaptic transistors hold tremendous potential for directly linking mechanical actions to artificial synaptic behaviors, enabling more realistic artificial neuromorphic devices. Yang et al. [27] introduced a multifunctional mechanoplastic artificial synapse that uses external displacement to achieve various synaptic plasticities. The mechanoplastic artificial synapse consists of an FG MoS_2_ synaptic transistor combined with TENG unit (Fig. 12A). The TENG displacement generates triboelectric potential, which couples with the gate of the FG synaptic transistor, acting as presynaptic voltage spikes. Meanwhile, the channel current realizes the functionalization of postsynaptic current signals (Fig. 12B). Accordingly, the synaptic weight can be easily adjusted by TENG displacement. Using the mechanoplastic artificial synapse, typical synaptic plasticity properties, such as P/D and paired-pulse depression/facilitation (PPD/F), were effectively emulated (Fig. 12C). Typically, the inhibitory/excitatory behavior of synapses can be enhanced with multiple pulses (Fig. 12D), where changes in inhibitory/excitatory postsynaptic current (IPSC/EPSC) represent enhanced inhibitory/excitatory behavior under multiple TENG displacement modulations. Taking inhibitory behavior as an example (Fig. 12E), as *D* increases, inhibitory gain decreases; under multiple mechanical displacement controls at 0.1 and 0.3 mm, inhibitory gain shows an increasing trend; however, no substantial change was observed under mechanical displacement control at 0.5 and 0.7 mm. This further proves that under larger displacements, the effect of consecutive multiple displacement pulses on *A_n_*/*A*_1_ gain is limited, and the artificial synapse can simultaneously achieve short-term and long-term plasticity, both of which are induced by mechanical displacements. Mechanoplastic artificial synapses realize active, self-powered adjustment of synaptic weight through direct mechanical behavior modification, displaying remarkable potential for application prospects in sensory synaptic fabrication, potential learning/memory stimulation, and advanced neuromorphic computing.

### Neuromorphic applications

Neuromorphic computing provides an effective pathway by incorporating analog memristors into ANNs or combining them with artificial neurons to build hardware-based neural networks (HW-NNs). While the memristor-based system offers a novel computational architecture, the development of artificial perceptual intelligence systems requires a great amount of supplementary analog and logic circuits [215–218]. Fortunately, the integration of synaptic devices with biometric sensors paves a new direction for advancing functional neuromorphic perceptual systems, commonly known as near-sensor (or edge) computing. In particular, the use of 2D FG synaptic devices to integrate memristors and sensors offers a novel route for multifunctional neuromorphic computing, especially for the development of artificial perceptual systems. Some key parameters of 2D-material-based FG devices and their neuromorphic applications are summarized in Table.

**Fig. 13.**
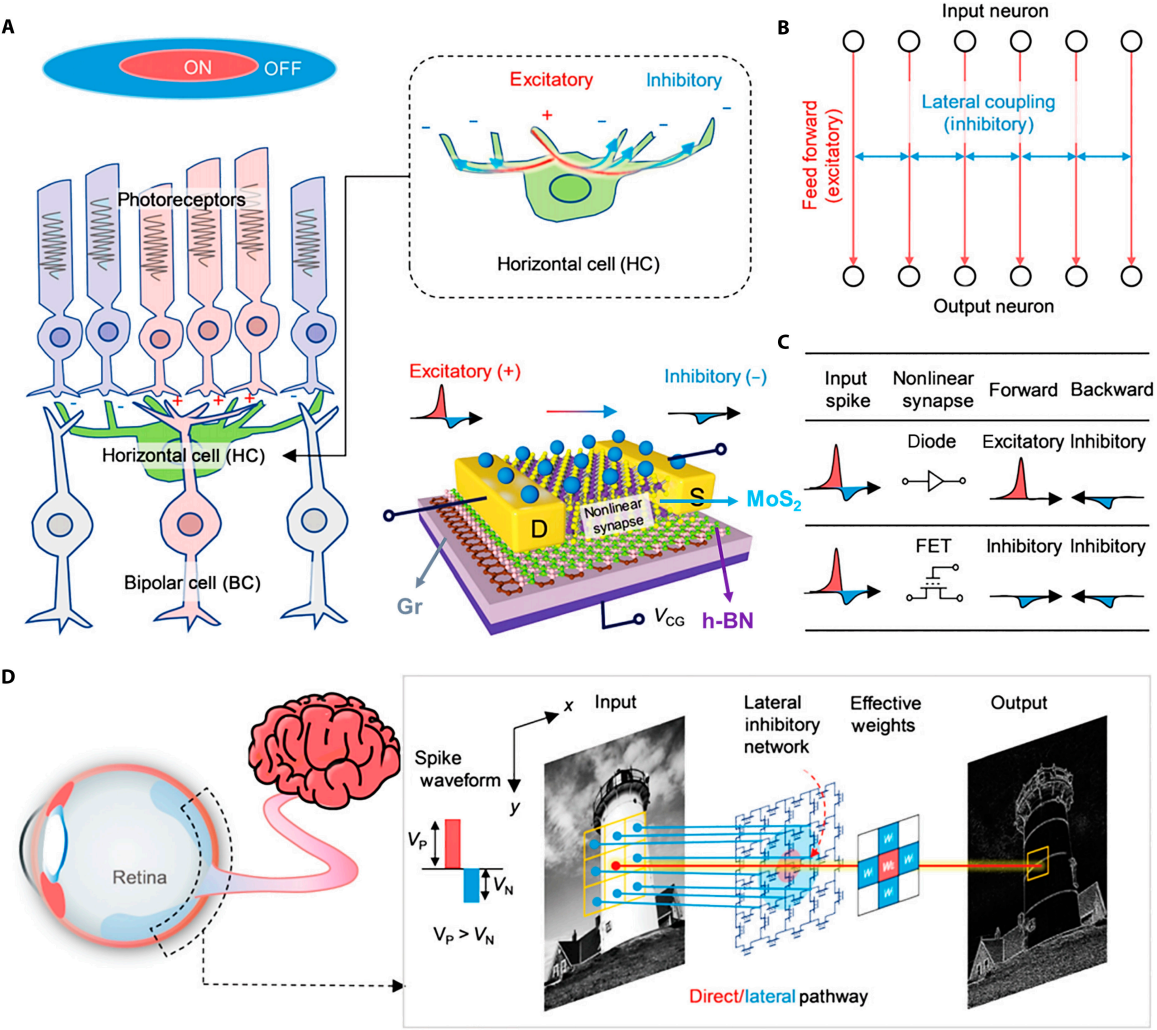
FG transistors based on 2D materials for retinal early visual processing. (A) Configuration of the FG synaptic transistor utilizing vdW heterostructures of the MoS_2_/h-BN/Gr and typical structure of the ON-center/OFF-surround receptive field in the retina. (B) A feedforward network with lateral inhibitory coupling can be formed using the bidirectional negative-pass rectification property of the threshold-tailored synaptic transistor, which distinguishes it from conventional diodes when subjected to a designed spike input. (C and D) Schematic of a 2D lateral network featuring the subthreshold FGFET, which enables basic retina-like edge enhancement through lateral inhibition [29].

**Fig. 14.**
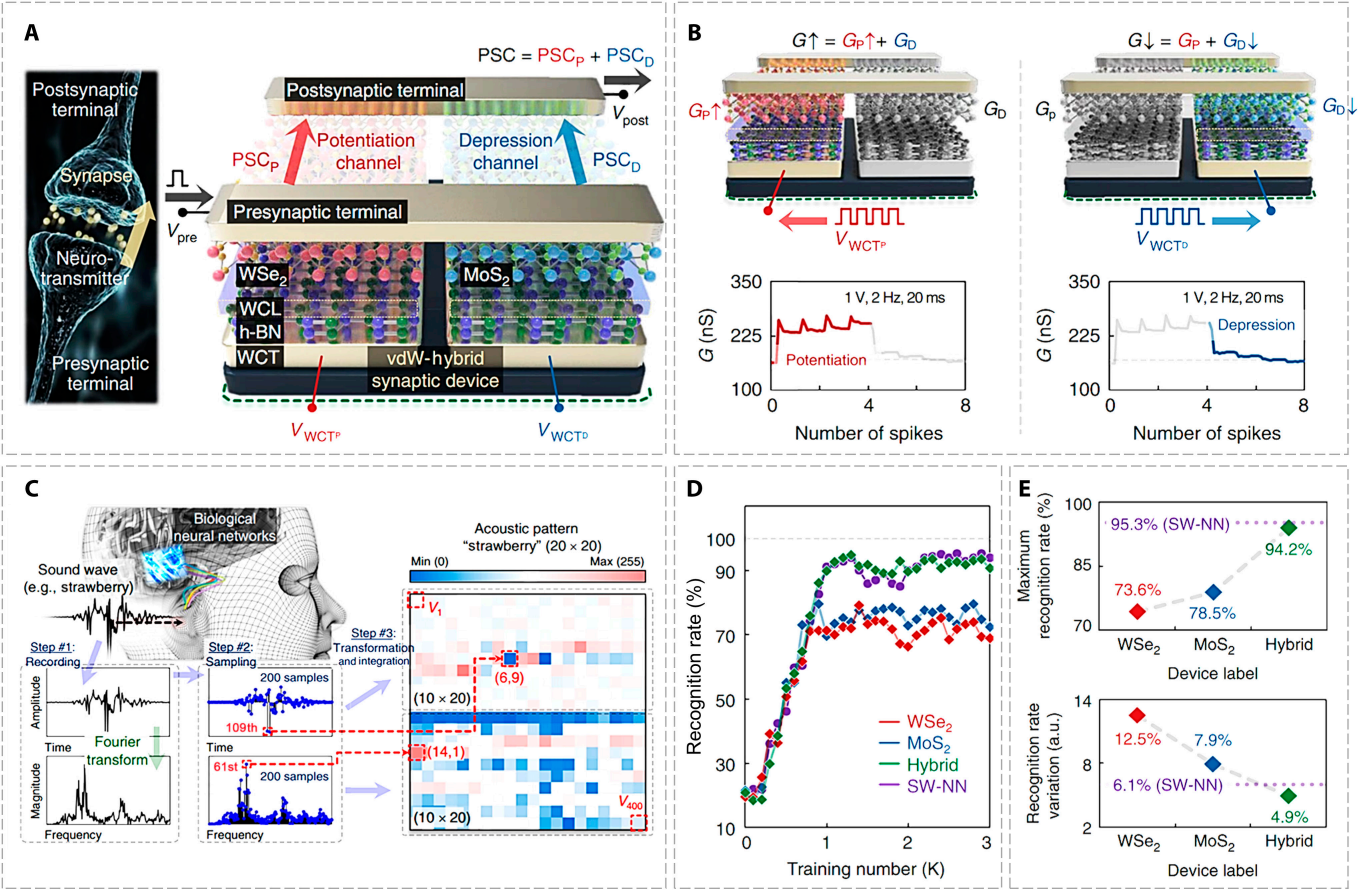
FG transistors based on 2D materials for auditory recognition. (A) A comparison of the functionality and structure between the biological synapse and the vdW-hybrid synaptic device. (B) Demonstration of P/D processes through the application of 4 spikes. (C) The process of designing acoustic pattern and designing 20 × 20 acoustic pattern, with each pixel displaying a grayscale value ranging from 0 to 255. (D) Acoustic pattern recognition rates were evaluated using 3 ANNs that incorporate WSe_2_, MoS_2_, or hybrid synaptic devices to compare with the rate achieved by SW-NN. (E) The maximum recognition rates and their associated variations [26]. a.u., arbitrary units.

**Fig. 15.**
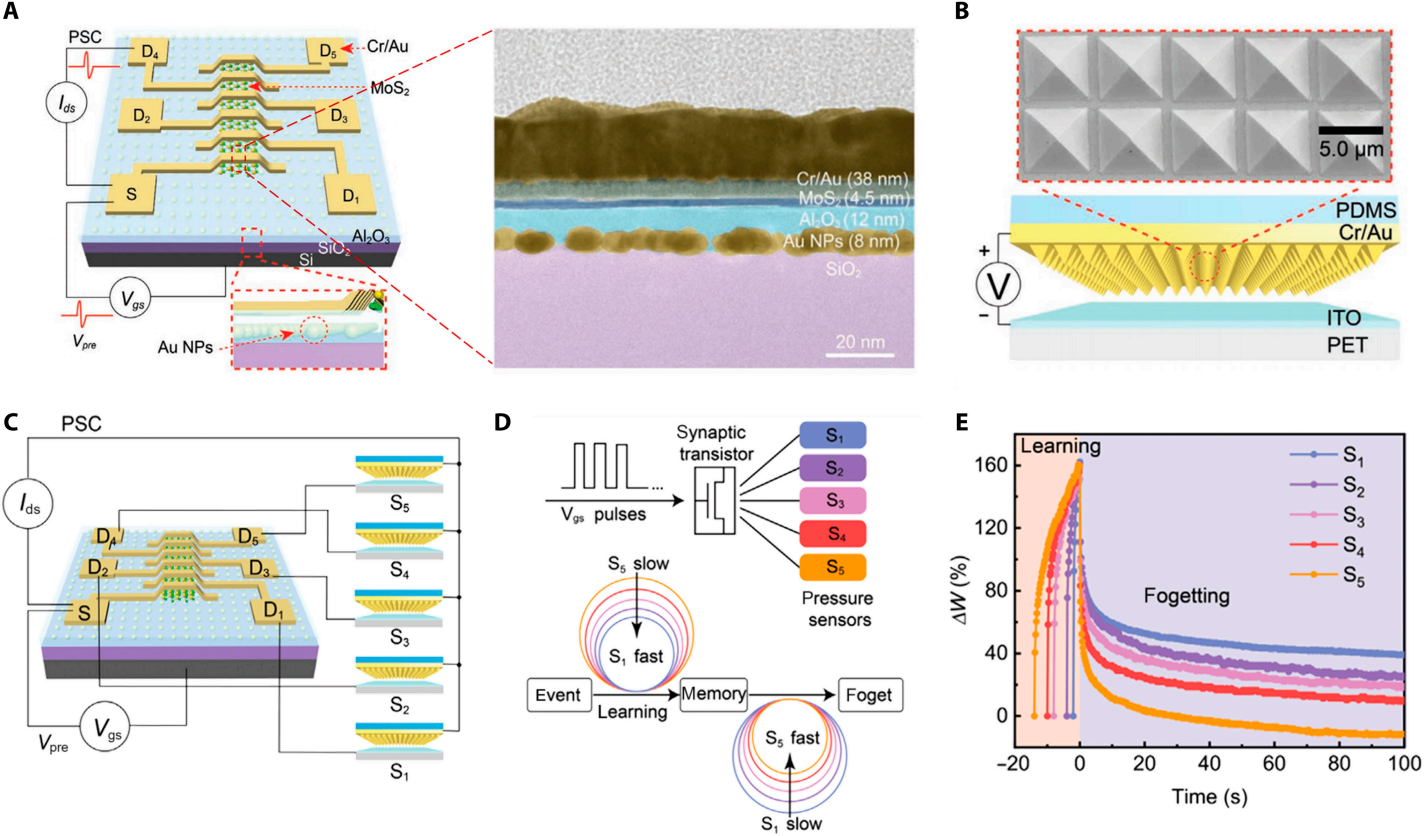
FG transistors based on 2D materials for tactile recognition. (A) Structure of MoS_2_ synaptic FG transistor (left) and its cross-sectional transmission electron microscopy image (right). (B) The scanning electron microscopy image (top) shows the PDMS film with distinct pyramid-shaped microstructures, while the bottom illustrates the structure of the PDMS pressure sensor. ITO, indium tin oxide; PET, polyethylene terephthalate. (C) The schematic of the integration of MoS_2_ FG synaptic transistor and PDMS pressure sensors. (D) Schematic of spatiotemporal modulation of synaptic plasticity (top) and learning and forgetting activity (bottom). (E) Simulation of the control of various learning and forgetting capabilities through touch interaction.

• Visual processing. Neuromorphic perception based on artificial synapses holds tremendous potential for low-energy and efficient processing of large volumes of continuous sensory data. From the perspective of neural network signal processing, the nonlinear coupling formed by synaptic connections between neurons ensures the unidirectional transmission of signals and plays a key role in noise reduction and feature extraction. Hu et al. [29] reported a bidirectional rectifying nonlinear synaptic device based on FG transistors. This transistor, composed of a MoS_2_/h-BN/graphene vdW heterostructure and utilizing ZnPc molecules as surface receptors, exhibits tunable nonlinear conductance, capable of bidirectionally modulating input pulse waveforms and allowing only excitatory or inhibitory currents to pass. Unlike conventional p-n junctions or Schottky junctions, which exhibit a specific rectification direction, the rectification in this subthreshold FG transistor arises from sensitive field-effect coupling between the drain bias and the FG potential. Rich synaptic plasticity, including spike-timing-dependent plasticity (STDP) and short-term plasticity (STP)/LTP, is achieved through charge injection to the FG. The nonlinear conductance of the artificial synapse not only lower pulse energy to 60 pJ but can also simulate the crucial lateral inhibition function by the horizontal retina cells. Fig. 13A shows the structure of the fabricated device and the structure of the central/peripheral receptive field structure in retinal bipolar cells. In this system, horizontal cells play a crucial role in forming on-center/off-periphery receptive fields by gathering excitatory signals from photoreceptors and delivering lateral inhibitory order to the bipolar cells. The fabricated FGFET’s negative transconductance and bidirectional rectification characteristics can be applied for lateral inhibitory coupling. When incorporated into a feed-forward network (Fig. 13B), it enables the lateral spread of inhibitory signals while forwarding excitatory signals. In contrast to other diode-based nonlinear synapses with specific rectification directions (Fig. 13C), the nonlinear synaptic transistor is essential for achieving bidirectional inhibitory coupling similar to horizontal cells under pulse inputs. A 2D network that incorporates the lateral coupling synapse (Fig. 13D) generates a receptive field with a weight distribution of center ON and periphery OFF, exhibiting edge enhancement functionality when integrated into a feed-forward network. Incorporating lateral coupling synapses represents a remarkable advancement in mimicking the early-stage information processing behaviors in biological systems, enabling the straightforward coupling of adjacent sensory units possible.
• Auditory recognition. Auditory recognition involves using synaptic devices to simulate the auditory processing functions of the human ear, converting sound signals into electrical signals, and processing and recognizing them through an HW-NN. However, HW-NNs utilizing these synaptic devices struggle to achieve the same accuracy to software-based neural networks (SW-NNs) for training and inference tasks. This limitation arises because the synaptic devices are not yet fully capable of meeting key characteristics such as device-to-device variation, cycle-to-cycle variation, endurance, retention characteristics, dynamic range, the number of conductance states, and the ability to provide linear/symmetric conductance changes [219–221]. In particular, the accuracy of inference following HW-NN training is greatly influenced by the linearity and symmetry of conductance changes. To overcome this challenge, Seo et al. [26] developed an FG hybrid synaptic device utilizing vdW heterostructures (Fig. 14A), successfully simulating biological synapses and exhibiting excellent synaptic characteristics. With linear and symmetric conductance updating properties, WSe_2_ and MoS_2_ channels were selectively applied for potentiation and depression conductance, achieving outstanding controllable conductance. Two signal paths for the P/D operation were realized by WSe_2_ (hole transport)/h-BN and MoS_2_ (electron transport)/h-BN vdW heterostructures. Because of the nondangling bond surface properties of 2D materials, vdW heterostructures do not cause lattice mismatch issues, thereby allowing the formation of FG structures without interface defects. The P/D channel was connected by 2 electrodes, and both had a separate gate electrode acting as a weight control terminal (WCT). Thus, the conductance (*G*) of the vdW hybrid synaptic device equals the sum of the conductance values of the potentiation (*G*_P_) and depression (*G*_D_) channels (*G* = *G*_P_ + *G*_D_). The synaptic conductance of this device could be enhanced or depressed by applying a positive pulse to the WCT (Fig. 14B). The feasibility to realizing HW-NN with vdW hybrid synaptic device was validated through simulations of both training and inference processes. By defining a method for converting sound signals into acoustic patterns (Fig. 14C), the acoustic pattern recognition rate of 3 types of ANNs can be obtained to compare with the recognition rate of SW-NN (Fig. 14D). The hybrid synaptic device achieved a recognition rate close to SW-NN, achieving a 94.2% recognition rate in the acoustic pattern recognition task with a minor variation rate of 4.9%. This study demonstrates the potential of constructing HW-NNs in high-precision brain-like computing.
• Tactile recognition. Artificial-synapse-based tactile perception systems offer efficient, low-energy advantages in health monitoring, bionic prostheses, and human–robot interface. Mo et al. [23] developed a multiterminal synaptic transistor that incorporates MoS_2_, with gold nanoparticles as the FG (Fig. 15A). By applying electric pulses to the gate, the synaptic transistor is capable of performing key biological synaptic functions. In addition, a pressure sensor with a pyramid microstructured polydimethylsiloxane (PDMS) was fabricated (Fig. 15B). By integrating the MoS_2_ synaptic transistor with the pressure sensor, an artificial tactile perception system was constructed (Fig. 15C), capable not only of identifying different touch locations—where touching different sensor locations results in different outputs due to the varying channel lengths connected to the sensors—but also of achieving spatial and temporal regulation of synaptic plasticity through modulation of different *V*_gs_ pulses and channel lengths. Furthermore, the system is capable of simulating the learning and forgetting behavior observed in the human brain. By applying a series of *V*_gs_ pulses, the weight change (∆*W*) can range from 0% to 160%, effectively simulating the repetitive learning process. Then, upon removal of the *V*_gs_ pulses, ∆*W* gradually decreases, simulating the forgetting process (Fig. 15D and E). By defining different levels of learning activity difficulties with various pressure sensors, it is possible to select learning efficiency/forgetting speed, successfully simulating the diverse learning/forgetting mode present in the human brain. This artificial synaptic system demonstrates an alternative interconnection scheme with strong synaptic weight adjustment capabilities, offering new insights for future neuromorphic sensory systems.

**Table.** Summary of the structure and properties of 2D-material-based FG transistors and their neuromorphic applications

Material	Device structure	Stimuli	Retention	Synaptic plasticity	Power consumption	Application	Reference
MoS_2_/h-BN/graphene	BFG	V-spikes	–	STP, LTP, and STDP	<60 pJ	Visual processing	[29]
vdW-hybrid	DFG	V-spikes	–	LTP and LTD	–	Auditory recognition	[26]
AuNPs/Al_2_O_3_/MoS_2_	BFG	V-spikes	–	PPF, STDP, and SRDP	–	Tactile recognition	[23]
MoS_2_/h-BN/graphene	TFG	V-spikes	~1,000 s	STM and LTM	–	Memory programming	[86]
ReS_2_/h-BN/graphene	BFG	V-spikes	–	PPF/D, STP, and LTP	0.06 nJ	Multitarget recognition	[92]
		L-spikes					
InSe/h-BN/graphene	BFG	M-spikes	>800 s	STP, STD, LTP, LTD, and PPF/D	165 aJ	Self-power synapse	[25]
MoS_2_/h-BN/graphene	BFG	V-spikes	–	STP, LTP, and PPF	2.52 fJ	Low-power synapse	[24]
		L-spikes					
MoS_2_/h-BN/graphene	2TFG	V-spikes	>10^4^	LTP and LTD	18 fJ	Neuromorphic computing	[20]
WSe_2_/h-BN/graphene	SFG	V-spikes	1,200 s	STM, LTM, STP, and LTP	74.2 fJ	Self-power synapse	[172]
		M-spikes					
MoS_2_/HfO_x_/HfS_2_	DFG	V-spikes	>5,000 s	SRDP, STDP, LTP, and LTD	–	Neuromorphic computing	[209]
MoS_2_/h-BN/graphene	EFG	V-spikes	–	STDP and PPF	0.31 pJ	Neuromorphic computing	[208]

BFG, bottom FG configurations; DFG, dual FG configurations; TFG, top FG configurations; V-spikes, voltage spikes; L-spikes, light spikes; M-spikes, mechanical spikes; STD, short-term depression; PPD, paired-pulse depression; PPF, paired-pulse facilitation; STM, short-term memory; LTM, long-term memory.

## Summaries and Outlook

**Fig. 16.**
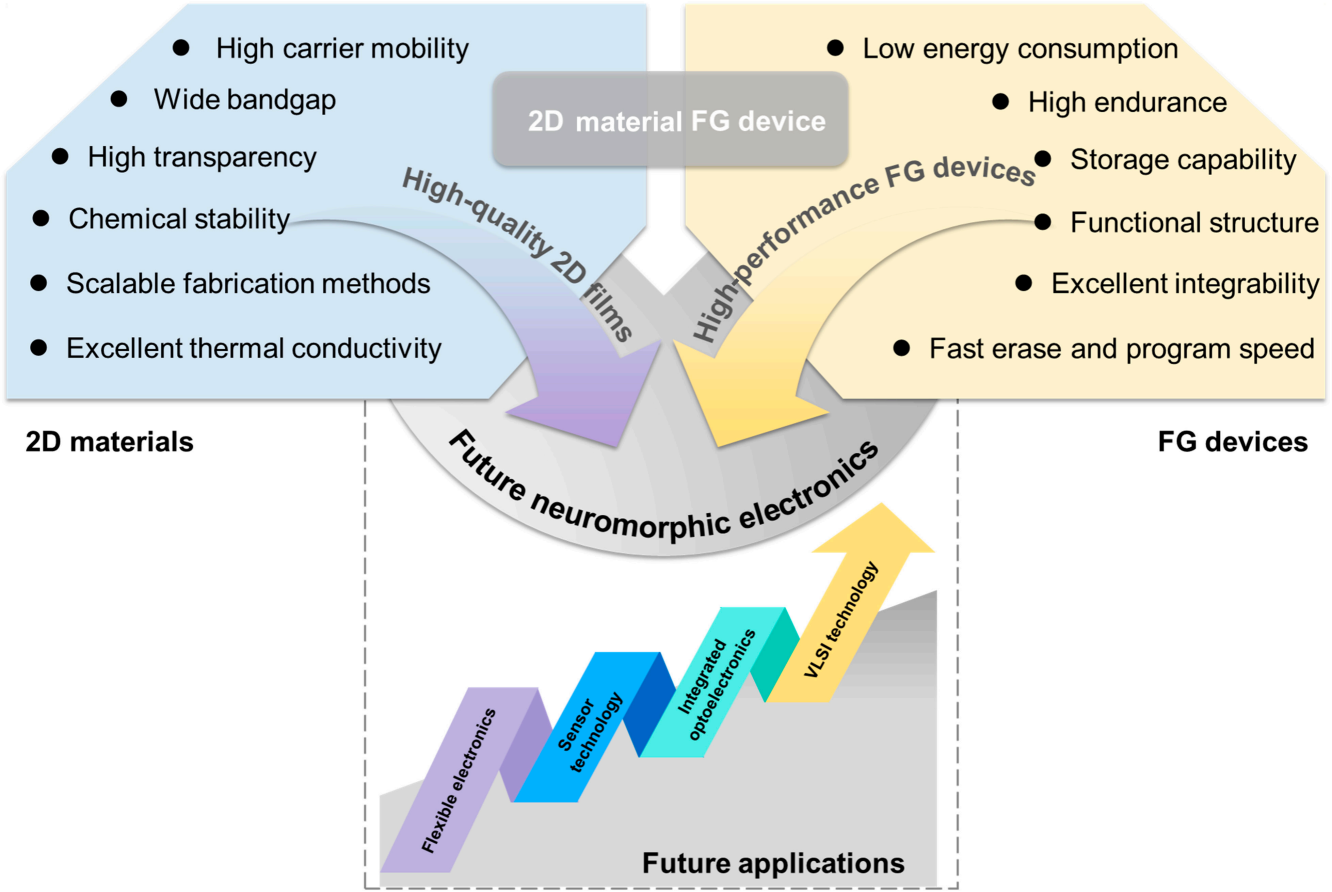
Summary and outlook of this review.

In this article, we explored the properties of typical 2D materials, the principles of FG transistors, and the applications of FG transistors incorporating 2D materials in memory, artificial synapses, and neuromorphic computing. A summary and outlook of this review are provided in Fig. 16. 2D materials, with their atomic-level thickness and unique electrical and optical properties, have shown great potential in micro/nanoelectronics [37–44]. FG transistors, an important class of nonvolatile memory devices, utilize the trapped charges in the FG to control the conductivity of the channel to realize the storage and retrieval of information [134]. The utilization of 2D materials has opened up new possibilities for FG transistors. By utilizing the distinctive properties of these materials, new FG devices and artificial synaptic devices with rapid response, low power consumption, and high stability have been developed.

In the domain of nonvolatile memory, 2D-material-based FG devices have demonstrated characteristics such as a high on/off ratio [90,209,222], ultrafast writing/erasing speeds [84,91,141,222,223], stable retention properties [24,85,144], and low power consumption [22,24,68,149]. Through various FG structures and modulation methods, a range of functionalities has been achieved, including nonvolatile storage [94,95,144,222,224], programmable p-n and n^+^-n junctions [30], logic rectification [94], broad-spectrum response [143], and photodetection, expanding the application scope of memory technology. In terms of artificial synapses, 2D FG devices can emulate the functions of biological neural synapses [LTP, LTD, STDP, and spike-rate-dependent plasticity (SRDP)], providing a foundation for building efficient ANNs and neuromorphic computing systems. Through electrical, optical, and mechanical modulation methods, precise control and adjustment of artificial synaptic performance have been achieved, providing a solid foundation for the future intelligent computing and sensing systems [20,24,92]. In the aspect of neuromorphic computing, FG devices utilizing 2D materials have shown application potential in visual processing, auditory recognition, tactile perception, and more [23,26,29]. By simulating the highly parallel computing and adaptive learning capabilities of the human brain, FG devices incorporating 2D materials hold the promise of overcoming the limitations of traditional von Neumann architecture, offering new solutions for processing large-dimensional data.

• Challenges of 2D-material-based FG devices. FG devices based on 2D materials also face technical bottlenecks, including issues related to material preparation, device structure design, device stability control, and integration. In the aspect of material preparation, obtaining large-area, high-quality, uniformly thick 2D material films remains a challenge. During the synthesis process of 2D crystals, nonuniformity and defect formation significantly influence device performance. The thickness of the crystal affects its electronic/optical/vibrational properties, making the thickness/uniformity of the material crucial for ensuring reliable device performance. A considerable portion of research has been dedicated to synthesizing materials with atomic-level thickness and large-scale lateral dimensions. Among these techniques, chemical vapor deposition is the most extensively studied [225], as it can produce uniform crystals with centimeter-level atomic thickness. However, its drawbacks include inconsistent growth rates and dynamics, as well as the need for high processing temperatures (typically >500 °C), which can lead to changes in film morphology or even defects.
• Explore new synthesis methods for 2D materials. Several methods have been reported, such as physical vapor deposition [226], atomic layer deposition [227], pulsed laser deposition [228,229], molecular beam epitaxy [230], and printing methods [231]. These methods offer new pathways for preparing large-area, high-quality 2D material crystals with precise thickness control, opening up possibilities for transparent, conductive, wide-bandgap semiconductor applications in large-area display panels and flexible, stretchable electronic products. Integrating printing methods, 2D materials enable the realization of miniaturized electronic components, reducing production costs and allowing for high customization and rapid production, offering new avenues for industrial development.
• Develop new device structure design. In addition to the typical FG structures mentioned, it is necessary to design more structures to integrate more functions into a single device while also considering device miniaturization. For instance, introducing new vertical or 3D stacked structural functional layers or edge contacts based on phase engineering could enhance device performance [95].
• Overcome stability and integration challenges. Regarding device stability control and integration, current research is mostly based on single devices. Reducing performance variability among devices and ensuring stability after integration is a major challenge. Achieving this objective requires addressing several challenges, such as reducing contact resistance, establishing reliable and controllable doping methods, enhancing carrier mobility, and optimizing the integration of high-κ dielectrics. In addition, the large-scale growth of uniform 2D material layers is essential to achieve low defect densities and minimal device-to-device deviations and maintain clean interfaces [65].
• Explore innovative applications for 2D materials FG devices. In future applications, 2D materials, with their rich band structure, offer more choices for FG device design, and the ultrathin characteristic brings benefits of device miniaturization, presenting significant advantages in very-large-scale integration (VLSI) applications. Easily constructing vdW heterostructures without concerns such as heterolattice mismatch in bulk semiconductors, 2D materials also possess unmatched surface-to-volume ratios, providing an inherent advantage responsive to surrounding physical/chemical stimuli. The sensitivity to external factors such as light, atmosphere, temperature, and humidity can undergo swift changes in surface electron transport. This sensitivity demonstrates fast response time and high sensitivities in sensors and integrated optoelectronic device domains [232]. Simultaneously, FG memories, leveraging the charge storage mechanism, can emulate synaptic plasticity, promising to overcome the traditional von Neumann computing architecture and serve as artificial synapses in brain-inspired neuromorphic computing [20–29].

Overall, 2D materials and FG transistor technology show broad application prospects in information storage, artificial synapses, and neuromorphic computing. In the future, we anticipate more extensive research and application of FG transistors integrated with 2D materials in fields such as flexible electronics, sensory technology, integrated optoelectronics, and VLSI, driving microelectronic technology toward higher efficiency, low energy consumption, and intelligence.

## Data Availability

All data needed to evaluate the conclusions in the paper are present in the paper and/or the Supplementary Materials. Additional data related to this paper may be requested from the authors.
